# Unexpected Tellurohalogenation of Terminal N‐Alkynyl (Alkenyl) Derivatives of 4‐Functionalized Pyrazoles

**DOI:** 10.1002/open.202400486

**Published:** 2025-01-26

**Authors:** Marianna V. Povidaichyk, Svitlana V. Shishkina, Eugeniy M. Ostapchuk, Mykhaylo Yu. Onysko

**Affiliations:** ^1^ Department of Uzhhorod national university 3 Narodna Sq Uzhhorod Ukraine; ^2^ Department of X-ray Diffraction Studies and Quantum Chemistry SSI “Institute for Single Crystals” NAS of Ukraine 60 Nauky ave. Kharkiv Ukraine; ^3^ Institute of Organic Chemistry NAS of Ukraine 5 Akademika Kuharya St Kyiv Ukraine; ^4^ Enamine Ltd 78 Winston Churchill St Kyiv Ukraine

**Keywords:** Alkynyl(alkenyl)pyrazole, Dihydropyrazolo[1.2-a]pyrazolium salts, Electrophilic heterocyclization, Tetrahydropyrazolo[1,2-a]pyridazinium salts, Zwitterion

## Abstract

The efficient synthesis of terminal N‐alkenyl and N‐alkynyl derivatives of pyrazole‐4‐carboxylic acids and their methyl esters as substrates for studying electrophilic heterocyclization has been reported. The regiochemistry and stereoselectivity of the tellurium‐induced heterocyclization of 1‐pentynyl (butynyl, butenyl) substituted pyrazole‐4‐carboxylic acids and their methyl esters under the action of tellurium (IV) oxide in hydrohalic acid were determined. This electrophilic heterocyclization leads to the formation of tetrahydropyrazolo[1,2‐a]pyridazinium and dihydropyrazolo[1,2‐a]pyrazolium intramolecular salts, as confirmed by X‐ray diffraction (XRD) and comprehensive spectral analysis.

## Graphical abstract

New tellurium‐containing tetrahydropyrazolo[1,2‐a]pyridazines and dihydropyrazolo[1,2‐a]pyrazoles were obtained by electrophilic heterocyclization in the form of intramolecular salts, and optimal conditions for their synthesis were found. The heterocyclization process is regio‐ and stereoselective, which is confirmed by spectral studies, and by X‐ray structural analysis. The mechanism of tellurium‐induced heterocyclization is proposed.

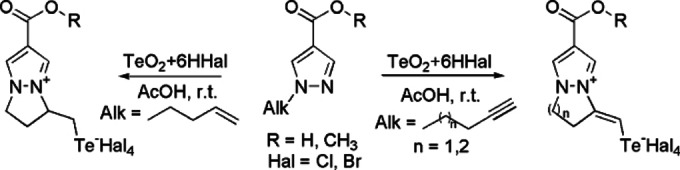

## Introduction

Developing general and cost‐effective methods for synthesizing analogues of natural biologically active compounds is highly attractive to synthetic chemists. Such analogues often demonstrate significant biological activity at a lower cost than structurally similar natural compounds.[^1^, ^2^] In particular, the natural alkaloids of Nigella species (nigelicine, nigelidine, nigeglapine, and nigeglaquine)[^3^, ^4^, ^5^] contain pyrazolo[1.2‐a]pyridazinium salts, which are the subject of our study.

**Figure 1:**
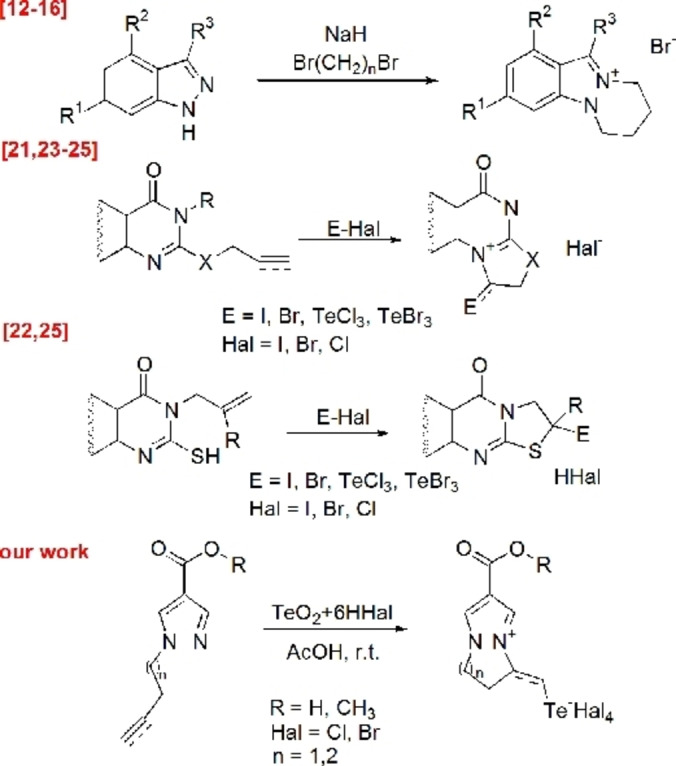
Synthetic approach to condensed salts of diazines.

Derivatives of this heterocyclic system exhibit a wide range of biological activities.[^5^, ^6^, ^7^, ^8^, ^9^, ^10^, ^11^] It is known that pyrazolo[1,2‐a]pyridazinium salts can be synthesized through the cyclization of functionalized indazole with dihaloalkanes or halogenoacyl halides[^12^, ^13^, ^14^, ^15^, ^16^] (Figure 1).

However, no data are currently available on the application of electrophilic intramolecular cyclization for the synthesis of pyrazolo[1,2‐a]pyridazines. Electrophilic heterocyclization of unsaturated substituted heterocycles is a convenient, versatile, and accessible method[^17^, ^18^, ^19^, ^20^] for synthesizing halogen‐ and chalcogen‐containing salts of heterocyclic compounds with diverse structures, including condensed salts of diazines[^21^, ^22^, ^23^, ^24^, ^25^] (Figure 1).

Based on our experience in the synthesis of fused aza‐heterocycles, we focused our studies with the aim of obtaining new potential biologically active derivatives of pyrazolo[1.2‐a]pyridazinium salts via electrophilic heterocyclization reactions. Several studies report[^26^, ^27^, ^28^, ^29^, ^30^, ^31^, ^32^, ^33^, ^34^, ^35^, ^36^] the potential of incorporating a tellurium‐halide fragment into organic molecules to produce compounds with valuable properties. This insight guided our choice of tellurium tetrahalides as the electrophilic reagent for the planned investigations.

## Results and Discussion

**Scheme 1:**
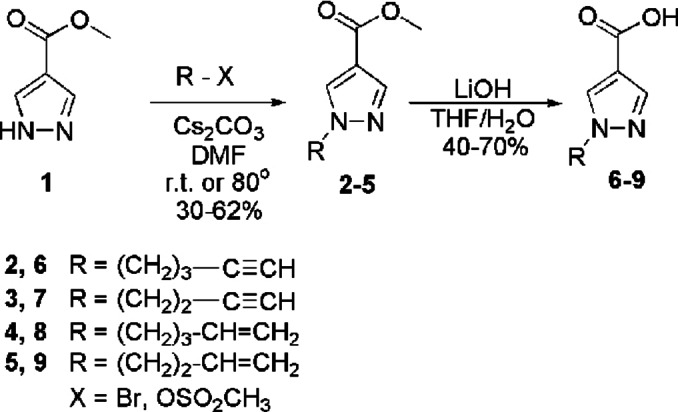
Synthesis of methyl 1‐alkynyl (alkenyl) pyrazole‐4‐carboxylates **2**–**5** and acids **6**–**9**.

The synthesis of the starting methyl esters of 1‐alkynyl (alkenyl) pyrazole‐4‐carboxylic acids 2–5 was performed by alkylation of methylpyrazolecarboxylate 1 with terminal alkenyl (alkynyl) halides or mesylates in the presence of cesium carbonate in dimethyl fomamide (DMF) medium. Acids 6–9 were obtained in moderate yields by alkaline hydrolysis of esters 2–5 using lithium hydroxide in a tetrahydrofuran (THF)/water medium, followed by acidification with sodium hydrogen sulfate (Scheme 1).

**Scheme 2:**
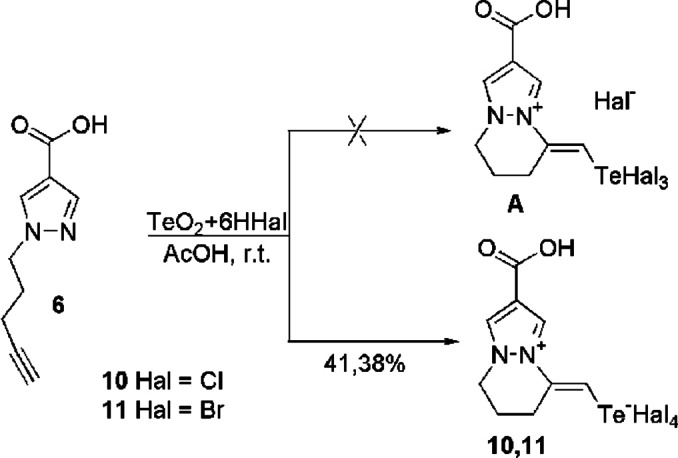
Synthesis of salts tetrahydropyrazolo [1.2‐a] pyridazinium **10**, **11**.

We used 1‐pentynylpyrazole‐4‐carboxylic acid **6** as an unsaturated substrate and tellurium tetrahalides as an electrophilic reagent (Table S1) to investigate the patterns of the electrophilic intramolecular cyclization reaction and identify the optimal synthesis conditions. According to the literature, tellurium tetrahalides can be utilized in such reactions either directly or *in situ* by generating them from tellurium dioxide and hydrohalogenide acid (HHal), which are in equilibrium with hydrogen hexahalogentellurate. The polarity of the solvent significantly influences this equilibrium mixture. When it interacts with an alkyl‐unsaturated azaheterocycle, the reaction can proceed via telluro‐induced[^22^, ^31^, ^34^, ^35^, ^36^] or proton‐induced[^30^, ^33^, ^37^] electrophilic cyclization, or via protonation of the primary center of the heterocycle, resulting in the formation of hexahalogentellurate salts.[^26^, ^27^, ^32^, ^38^] It should be noted that the regiochemistry of the process depends on the reaction conditions and the nature of the azaheterocycle. We subjected 1‐pentynylpyrazole‐4‐carboxylic acid **6** to the action of an equilibrium mixture formed *in situ* from tellurium dioxide and an excess of hydrohalogenide acid under different reaction conditions (time, temperature, ratio of reagents, solvent). As a result, we found the optimal conditions for achieving the maximum yield of the target reaction products **10**, **11** (Scheme 2). The optimal conditions are as follows: solvent – glacial acetic acid (AcOH), the ratio of reagents – acid **6**: tellurium dioxide: hydrohalogenide acid=1 : 1 : 6; reaction temperature −20 °C, reaction time −24 hours (Table S1).

**Figure 2:**
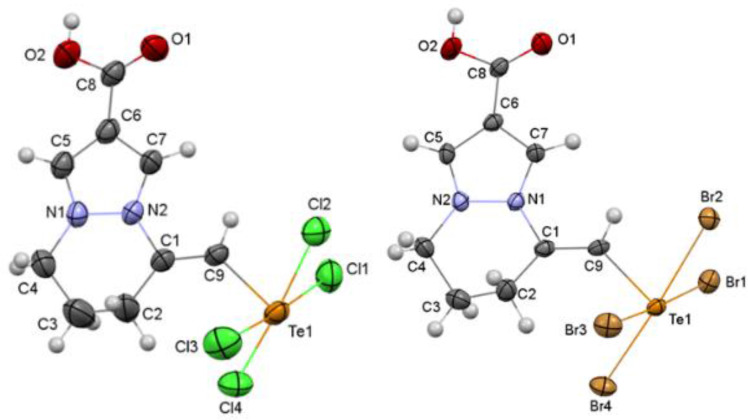
Molecular structure of compounds **10** (on the left) and **11** (on the right). Thermal ellipsoids of non‐hydrogen atoms are shown at 50 % probability level.

We investigated the obtained cyclization products **10**, **11** using a complex of spectral analysis methods. The occurrence of tellurium‐induced cyclization was confirmed by the proton signal of the telluromethylidene group in the ^1^H NMR spectrum of product **10** which appeared as a singlet in a weak field at 8.11 ppm. In the ^13^C NMR spectrum, decisive signals included those of the telluromethylidene group's carbon at 131.9 ppm and the C5 carbon of the pyridazine ring at 139.3 ppm. The spectral characteristics of salt **11** are similar to **10**, which also indicated the formation of analogical structure of the target product **11**. This allows us to assert, that the nature of the halogen had minimal influence on the cyclization process, as evidenced by only slight differences in yields. Based on analogy with previous products of tellurium‐induced cyclization,[^22^, ^31^, ^34^, ^35^] we initially assigned the structure of salts **10**, **11** as a tetrahydropyrazolo[1.2‐a]pyridazinium cations with a trihalomethylidene group and a halogen anion (Scheme 2, structure **A**), consistent with prior studies on tellurium‐induced heterocyclization. However, X‐ray structural studies of salts **10**, **11** clearly indicated the formation of a zwitterion with the *E*‐configuration of the tellurium‐containing group (Figure 2). This formation, unlike any previously described for electrophilic heterocyclization products, had not been reported for any heterocyclic system.

**Scheme 3:**
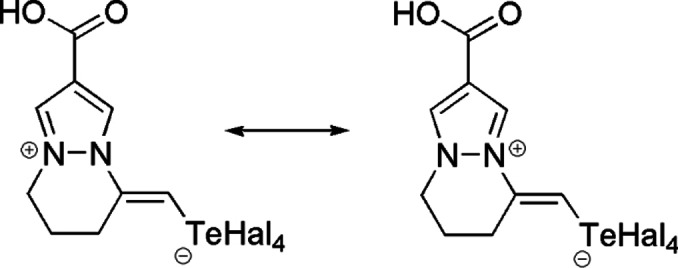
Resonance structures of compounds **10** and **11**.

Analysis of the molecular structure of compounds **10** and **11** showed that the lengths of the C5−C6 and C6−C7 bonds, as well as N1−C5 and N2−C7 bonds, are very similar in both molecules (Table 1). The Te atom, formally tetravalent, is bonded to the C9 atom and four halogen atoms. Consequently, the structure of compounds **10** and **11** can be described as a superposition of two resonance zwitterionic structures (Scheme 3). The four Te−Hal bonds are not equivalent, the Te1−Cl2/Br2 bond is slightly longer than the other three (Table 1). However, this non‐equivalence arises not only to the localization of the negative charge, but also to the involvement of the halogen atom in the intermolecular hydrogen bond as a proton acceptor. In the structure of **10**, this is evidenced by the hydrogen bond O2−H…Cl’ (symmetry operation: 1‐x, 1‐y, ‐z), with an H…Cl distance of 2.43 Å and an O−H…Cl angle of 153°. Similarly, in the structure of **11**, the hydrogen bond O2−H…Br’ (symmetry operation: 1‐x, 1‐y, ‐z) has an H…Br distance of 2.56 Å and an O−H…Br angle of 144°. According to the theory of σ‐hole interactions, a small region of positive charge of the Te atom is located on the continuation of the C9−Te1 bond. The orientation of this region relative to the halogen atom of the neighboring molecule (symmetry operation is 2‐x,‐y,1‐z; the angle C9−Te1…Hal is 165.8(1)° in the structure **10** or 170.8(1)° in the structure **11)** and the distance Te…Hal (3.695(1) Å in the structure **10** or 3.6073(6) Å in the structure **11**, shorter than the corresponding the van der Waals radii sums^[39]^ (3.94 Å for the Te…Cl distance and 4.08 Å for the Te…Br distance), allow us to assert the presence of chalcogen intermolecular interactions in the crystal.

**Table 1:** Some bond lengths (in Å) in molecules **10** and **11** according to X‐ray diffraction data.

Bond length	Molecule 10	Molecule 11
C5−C6	1.374 (7)	1.371 (9)
C6−C7	1.374 (5)	1.363 (6)
N1−C5	1.333 (6)	1.334 (7)
N2−C7	1.338 (7)	1.346 (9)
Te1−C1	2.539 (2)	2.7364 (8)
Te1−Cl2	2.604 (1)	2.7659 (7)
Te1−Cl3	2.466 (2)	2.6302 (8)

The partially saturated six‐membered heterocycle adopts a half‐chair conformation in both molecules. The C1, N2, N1, C4 atoms lie in the plane while the deviations of the C2 and C3 atoms from this plane are −0.182(10) Å and 0.444(12) Å, respectively, in molecule **10** and −0.242(9) Å and 0.491(10) Å, respectively, in molecule **11**. The tellurium‐containing group adopts a *cis*‐conformation to the C1−C2 endocyclic bond, with the C2−C1−C9−Te1 torsion angle measuring 3.3(7)° in molecule **10** and 1.4(9)° in molecule **11**.

**Scheme 4:**
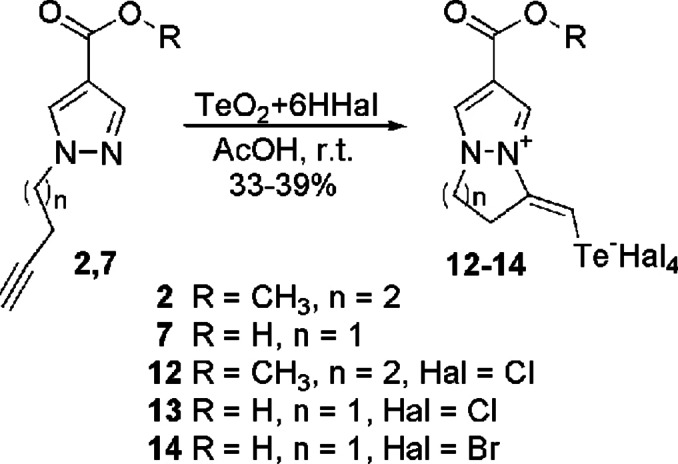
Synthesis of aza‐annelated salts **12**–**14**.

The identified direction and optimal conditions for the tellurohalogenation reaction were successfully applied to other terminal alkynyl derivatives of 4‐functionalized pyrazole, namely methyl 1‐pentynylpyrazole‐4‐carboxylate **2** and 1‐butynylpyrazole‐4‐carboxylic acid **7** (Scheme 4). According to spectral data, tellurium‐induced heterocyclization occurs in all cases, resulting in the formation of halotelluromethylidene‐substituted tetrahydropyrazolo [1,2‐a] pyridazinium salts **12** and dihydropyrazolo [1,2‐a] pyrazolium **13**, **14**. The size of the ring fused to the pyrazole is influenced by the length of the alkynyl substituent, while the chemical shift of the proton in the telluromethylidene group in the ^1^H NMR spectra confirms the formation of compounds **12**–**14** with an *E*‐configuration.

**Scheme 5:**
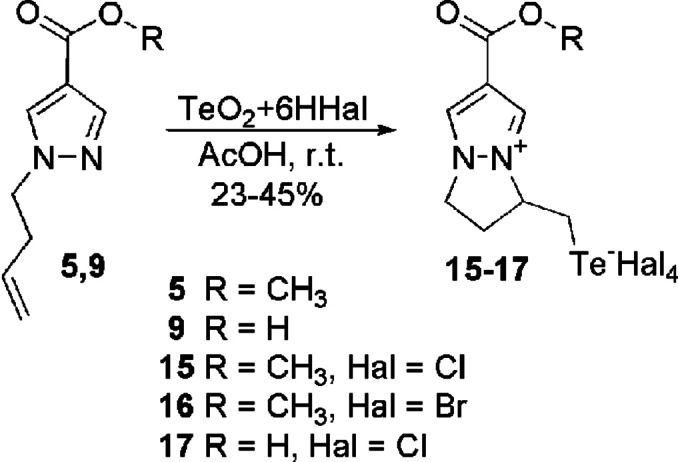
Synthesis of dihydropyrazolo [1.2‐a] pyrazolium salts **15**–**17**.

The regiochemistry of the observed tellurium‐induced heterocyclization was also confirmed for terminal N‐butenyl derivatives of 4‐functionalized pyrazole 5, 9 (Scheme 5). Ester 5 and acid 9, along with their alkynyl analogues, were treated under the same conditions with tellurite tetrahalides obtained *in situ* in acetic acid. As a result, telluromethyl‐substituted dihydropyrazolo [1.2‐a] pyrazolium salts[^15^, ^16^, ^17^] were synthesized in moderate yields.

**Scheme 6:**
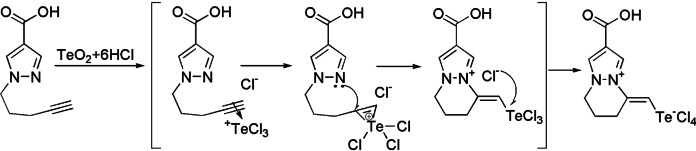
Plausible mechanism of tellurium‐induced cyclization of acid **6**.

To explain the unexpected formation of internal salts,[^10^, ^11^, ^12^, ^13^, ^14^, ^15^, ^16^, ^17^] we propose the following mechanism for their synthesis, using the cyclization of compound 6 as an example. The process begins with the action of the electrophilic reagent (trihalogenotelluronium cation) on the triple bond of the pentynyl substituent, leading to the formation of a three‐center telluronium cation (Scheme 6). Subsequently, intramolecular cyclization occurs at the N2 nitrogen of the pyrazole, followed by a Lewis acid‐base interaction that results in the formation of a zwitterion. It should be noted that the proposed mechanism supports the formation of the product in the *E*‐configuration.

## Conclusions

A simple and accessible method was developed, along with optimal conditions, for the synthesis of tellurium‐containing salts of tetrahydropyrazolo [1.2‐a] pyridazinium and dihydropyrazolo [1.2‐a] pyrazolium, as analogues of natural alkaloids: solvent – glacial acetic acid (AcOH), the ratio of reagents – acid 6: tellurium dioxide: hydrohalogenide acid=1 : 1 : 6; reaction temperature −20 °C, reaction time −24 hours. It was found that the heterocyclization process is regio‐ and stereoselective, leading to the unexpected formation of intramolecular salts, the structure of which has been thoroughly investigated using a combination of spectral methods and X‐ray structural analysis. The obtained condensed salts are promising objects for studying their biological activity.

## Experimental Part

^1^H, ^13^C NMR spectra were recorded on a Varian Unity Plus 400 spectrometer (400 MHz for protons, 101 MHz for carbon‐13) in dimethyl sulfoxide‐d_6_ ([D^6^] DMSO). Tetramethylsilane (^1^H, ^13^C) was used as a standard. Melting points were determined using a Stuart SMP30 instrument. Elemental analyzes were performed using an Elementar Vario MICRO cube analyzer. All reagents were procured from commercial suppliers and used without further purification. Anhydrous solvents were prepared following standard procedures.

Methyl 1‐(pent‐1‐yn‐5‐yl)‐1H‐pyrazole‐4‐carboxylate **2**. To 0.016 mol of methyl pyrazole‐4‐carboxylate 1 dissolved in 20 ml of DMF, add 0.24 mol of cesium carbonate and stir for 30 minutes. Add 0.0176 mol of pentinyl mesylate to the suspension. The reaction mixture was stirred for 8 hours at 80 °C. The resulting solution is poured into water, extracted with ethyl acetate, evaporated and a brown liquid is obtained. Yield 52 %. ^1^H NMR (400 MHz, [D^6^] DMSO): δ=1.96 (m, 2H, CH_2_), 2.12 (m, 2H, CH_2_), 2.83 (s, 1H, ≡CH), 3.73 (s, 3H, OCH_3_), 4.21 (t, J=8.0 Hz, 2H, NCH_2_), 7.86 (s, 1H, CH_pyrazol_), 8.33 (s, 1H, CH_pyrazol_); ^13^C NMR (101 MHz, DMSO D^6^): δ=15.4 (CH_2_), 28.9 (CH_2_), 50.9 (NCH_2_), 51.5 (OCH_3_), 72.2 (≡CH), 83.5 (−C≡), 114.0 (C–COOCH_3_), 134.2 (CH_pyrazol_), 140.8 (CH_pyrazol_), 163.2 (COOCH_3_); elemental analysis calcd (%) for C_10_H_12_N_2_O_2_: C 62.49, H 6.29, N 14.57; found: C 62.56, H 6.54, N 14.31.

### General Procedure for Synthesis of Methyl 1‐Alkynyl (Alkenyl) Pyrazole‐4‐Carboxylates 3–5

To 0.016 mol of methyl pyrazole‐4‐carboxylate 1, dissolved in 20 ml of DMF, add 0.24 mol of cesium carbonate and stir for 30 minutes. Add 0.0176 mol of alkyl (butynyl, butenyl, pentenyl) bromide to the suspension. The reaction mixture was stirred for 8 hours at room temperature. The resulting solution is poured into water, extracted with ethyl acetate, evaporated and a brown liquid is obtained.

Methyl 1‐(but‐1‐yn‐4‐yl)‐1H‐pyrazole‐4‐carboxylate 3. Yield 30 %. ^1^H NMR (400 MHz, [D^6^] DMSO): δ=2.72 (m, 2H, CH_2_), 2.87 (s, 1H, ≡CH), 3.74 (s, 3H, OCH_3_), 4.27 (t, J=6.6 Hz, 2H, NCH_2_), 7.88 (s, 1H, CH_pyrazol_), 8.37 (s, 1H, CH_pyrazol_). ^13^C NMR (101 MHz, DMSO D^6^): δ=19.4 (CH_2_), 50.2 (NCH_2_), 51.1 (OCH_3_), 73.1 (≡CH), 80.7 (−C≡), 113.4 (C–COOCH_3_), 133.9 (CH_pyrazol_), 140.4 (CH_pyrazol_), 162.7 (COOCH_3_); elemental analysis calcd (%) for C_9_H_10_N_2_O_2_: C 60.66, H 5.66, N 15.72; found: C 61.01, H 5.48, N 15.64.

Methyl 1‐(pent‐1‐en‐5‐yl)‐1H‐pyrazole‐4‐carboxylate 4. Yield 62 %. ^1^H NMR (400 MHz, [D^6^] DMSO): δ=1.87 (m, 2H, CH_2_), 1.96 (m, 2H, CH_2_), 3.73 (s, 3H, OCH_3_), 4.14 (t, J=6.8 Hz, 2H, NCH_2_), 5.00 (dd, J=19.2, J=11.2, 2H, =CH_2_), 5.79 (m, 1H, ≡CH−), 7.86 (s, 1H, CH_pyrazol_), 8.35 (s, 1H, CH_pyrazol_). ^13^C NMR (101 MHz, DMSO D^6^): δ=28.6 (CH_2_), 29.9 (CH_2_), 50.9 (NCH_2_), 51.2 (OCH_3_), 114.5 (C–COOCH_3_), 115.4 (=CH_2_), 133.5 (CH_pyrazol_), 137.4 (−CH=), 140.3 (CH_pyrazol_), 163.7 (COOCH_3_); elemental analysis calcd (%) for C_10_H_14_N_2_O_2_: C 61.84, H 7.27, N 14.42; found: C 62.05, H 7.10, N 14.33.

Methyl 1‐(but‐1‐en‐4‐yl)‐1H‐pyrazole‐4‐carboxylate 5. Yield 46 %. ^1^H NMR (400 MHz, [D^6^] DMSO): δ=2.55 (m, 2H, CH_2_), 3.73 (s, 3H, OCH_3_), 4.22 (t, J=6.8 Hz, 2H, NCH_2_), 5.00 (dd, J=16.8 Hz, J=10 Hz, 2H, =CH_2_), 5.73 (m, 1H, =CH−), 7.85 (s, 1H, CH_pyrazol_), 8.33 (s, 1H, CH_pyrazol_). ^13^C NMR (101 MHz, DMSO D^6^): δ=33.6 (CH_2_), 50.8 (NCH_2_), 51.0 (OCH_3_), 114.4 (C–COOCH_3_), 117.3 (=CH_2_), 133.6 (CH_pyrazol_), 134.5 (−CH=), 140.1(CH_pyrazol_), 158.5 (COOCH_3_); elemental analysis calcd (%) for C_9_H_12_N_2_O_2_: C 59.99, H 6.71, N 15.55; found: C 60.17, H 6.63, N 15.48.

### General Procedure for Synthesis of 1‐Alkynyl (Alkenyl) Pyrazole‐4‐Carboxylic Acids 6–9

To 0.026 mol of methyl 1‐alkynyl (alkenyl) pyrazole‐4‐carboxylate 2–5, dissolved in 10 ml of THF, add 0.26 mol of LiOH in 10 ml of water. The reaction mixture is stirred for 48 hours. THF is evaporated, the solution is acidified with aqueous sodium hydrogen sulfate, and the formed precipitate is filtered, dried in air.

1‐(Pent‐1‐yn‐5‐yl)‐1H‐pyrazole‐4‐carboxylic acid 6. Yield 56 %. m.p. 106–108 °C ^1^H NMR (400 MHz, [D^6^] DMSO): δ=1.93 (m, 2H, CH_2_), 2.12 (m, 2H, CH_2_), 2.83 (s, 1H, ≡CH), 4.20 (t, J=7.0 Hz, 2H, NCH_2_), 7.80 (s, 1H, CH_pyrazol_), 8.23 (s, 1H, CH_pyrazol_), 12.28 (s, 1H, COOH). ^13^C NMR (101 MHz, DMSO D^6^): δ=14.9 (CH_2_), 28.4 (CH_2_), 50.3 (NCH_2_), 71.8 (≡CH), 83.1 (−C≡), 114.7 (C–COOH), 133.6 (CH_pyrazol_), 140.5 (CH_pyrazol_), 163.7 (COOH); elemental analysis calcd (%) for C_9_H_10_N_2_O_2_: C 60.66, H 5.66, N 15.72; found: C 60.79, H 5.66, N 15.67.

1‐(But‐1‐yn‐4‐yl)‐1H‐pyrazole‐4‐carboxylic acid 7. Yield 40 %. m.p. 104–105 °C ^1^H NMR (400 MHz, [D^6^] DMSO): δ=2.71 (m, 2H, CH_2_), 2.85 (s, 1H, ≡CH), 4.26 (t, J=6.6 Hz, 2H, NCH_2_), 7.81 (s, 1H, CHpyrazol), 8.27 (s, 1H, CH_pyrazol_), 12.31 (s, 1H, COOH); elemental analysis calcd (%) for C_8_H_8_N_2_O_2_: C 58.53, H 4.91, N 17.06; found: C 58.47, H 4.85, N 17.11.

1‐(Pent‐1‐en‐5‐yl)‐1H‐pyrazole‐4‐carboxylic acid 8. Yield 67 %. m.p. 92–94 °C ^1^H NMR (400 MHz, [D^6^] DMSO): δ=1.86 (m, 2H, CH_2_), 1.96 (m, 2H, CH_2_), 4.13 (t, J=6.8 Hz, 2H, NCH_2_), 5.00 (dd, J=20 Hz, J=12 Hz, 2H, =CH_2_), 5.81 (m, 1H, =CH−), 7.89 (s, 1H, CH_pyrazol_), 8.24 (s, 1H, CH_pyrazol_), 11.76 (s, 1H, COOH); elemental analysis calcd (%) for C_9_H_12_N_2_O_2_: C 59.99, H 6.71, N 15.55; found: C 60.07, H 6.64, N 15.48.

1‐(But‐1‐en‐4‐yl)‐1H‐pyrazole‐4‐carboxylic acid 9. Yield 70 %. m.p. 115–117°C ^1^H NMR (400 MHz, [D^6^] DMSO): δ=2.55 (m, 2H, CH_2_), 4.20 (t, J=6.8 Hz, 2H, NCH_2_), 5.01 (dd, J=16 Hz, J=10.4 Hz, 2H, =CH_2_), 5.74 (m, 1H, =CH−), 7.78 (s, 1H, CH_pyrazol_), 8.22 (s, 1H, CH_pyrazol_), 12.26 (s, 1H, COOH). ^13^C NMR (101 MHz, DMSO D^6^): δ=33.7 (CH_2_), 50.8 (NCH_2_), 114.4 (C–COOH), 117.3 (=CH_2_), 133.5 (CH_pyrazol_), 134.6 (−CH=), 140.3 (CH_pyrazol_), 163.7 (COOH); elemental analysis calcd (%) for C_8_H_10_N_2_O_2_: C 57.82, H 6.07, N 16.86; found: C 57.96, H 5.95, N 16.77.

### General Procedure for Synthesis of Salts 10–17

A solution of tellurium tetrahalide formed from 0.0017 mol of tellurium dioxide and 0.0102 mol of concentrated hydrohalogenide acid is added dropwise to 0.0017 mol of acid 6–9 or ester 2–5 dissolved in 10 ml of glacial acetic acid. The reaction mixture is stirred at room temperature for 24 hours, the formed precipitate is filtered, dried in air.

(5 E)‐2‐carboxy‐5‐[(tetrachloro‐λ5‐tellanyl) methylene]‐5,6,7,8‐tetrahydropyrazolo [1,2‐a] pyridazin‐4‐ium 10. Yield 41 %. m.p. 240 °C ^1^H NMR (400 MHz, [D^6^] DMSO): δ=2.18 (m, 2H, CH_2_), 3.54 (m, 2H, CH_2_), 4.56 (m, 2H, NCH_2_), 8.11 (s, 1H, =CHTe), 9.19 (s, 1H, CH_pyrazol_), 9.80 (s, 1H, CH_pyrazol_), 13.81 (s, 1H, COOH). ^13^C NMR (101 MHz, DMSO D^6^): δ=18.4 (CH2), 24.4 (CH_2_), 50.2 (NCH_2_), 116.9 (C–COOH), 131.9 (=CHTe−Cl4), 134.5 (CH_pyrazol_), 139.3 (=CN^+^), 139.8 (CH_pyrazol_), 160.7 (COOH); elemental analysis calcd (%) for C_9_H_10_C_l4_N_2_O_2_Te: C 24.15, H 2.25, Cl 31.68, N 6.26; found: C 24.21, H 2.28, Cl 31.60, N 6.24.

(5 E)‐2‐carboxy‐5‐[(tetrabromo‐λ5‐tellanyl) methylene]‐5,6,7,8‐tetrahydropyrazolo [1,2‐a] pyridazin‐4‐ium 11. Yield 38 %. m.p. 252 °C ^1^H NMR (400 MHz, [D^6^]DMSO): δ=2.21 (m, 2H, CH_2_), 3.56 (m, 2H, CH_2_), 4.58 (m, 2H, NCH_2_), 8.21 (s, 1H, =CHTe), 9.20 (s, 1H, CH_pyrazol_), 9.76 (s, 1H, CH_pyrazol_). ^13^C NMR (101 MHz, DMSO D^6^): δ=18.4 (CH2), 26.0 (CH_2_), 50.2 (NCH_2_), 116.9 (C–COOH), 120.5 (=CHTe‐Br_4_), 134.4 (CH_pyrazol_), 138.4 (=CN^+^), 139.8 (CH_pyrazol_), 160.7 (COOH); elemental analysis calcd (%) for C_9_H_10_Br_4_N_2_O_2_Te: C 17.28, H 1.61, Br 51.11, N 4.48; found: C 17.37, H 1.56, Br 51.17, N 4.43.

(5 E)‐2‐(methoxycarbonyl)‐5‐[(tetrachloro‐λ5‐tellanyl) methylene]‐5,6,7,8‐tetrahydropyrazolo [1,2‐a] pyridazin‐4‐ium 12. Yield 33 %. m.p. 210–212 °C ^1^H NMR (400 MHz, [D^6^] DMSO): δ=2.19 (m, 2H, CH_2_), 3.55 (m, 2H, CH_2_), 3.89 (s, 3H, OCH_3_), 4.57 (t, J=6 Hz, 2H, NCH_2_), 8.15 (s, 1H, =CHTe), 9.30 (s, 1H, CH_pyrazol_), 9.95 (s, 1H, CH_pyrazol_). ^13^C NMR (101 MHz, DMSO D^6^): δ=18.9 (CH_2_), 24.9 (CH_2_), 50.9 (NCH_2_), 53.1 (OCH_3_), 117.0 (C–COOCH_3_), 132.5 (=CHTe‐Cl_4_), 135.1 (CH_pyrazol_), 139.8 (=CN^+^), 140.2 (CH_pyrazol_), 160.4 (COOCH_3_); elemental analysis calcd (%) for C_10_H_12_Cl_4_N_2_O_2_Te: C 26.02, H 2.62, Cl 30.72, N 6.07; found: C 26.12, H 2.71, Cl 30.68, N 6.01.

(3 E)‐6‐carboxy‐3‐[(tetrachloro‐λ5‐tellanyl) methylene]‐2,3‐dihydro‐1H‐pyrazolo [1,2‐a] pyrazol‐4‐ium 13. Yield 39 %. m.p. 225–226 °C ^1^H NMR (400 MHz, [D^6^] DMSO): δ=4.22 (t, J=6.4 Hz, 2H, CH_2_), 4.65 (t, J=6.8 Hz, 2H, NCH_2_), 8.18 (s, 1H, =CHTe), 9.15 (s, 1H, CH_pyrazol_), 9.95 (s, 1H, CH_pyrazol_), 13.78 (s, 1H, COOH). ^13^C NMR (101 MHz, DMSO D^6^): δ=29.5 (CH_2_), 48.2 (NCH_2_), 117.7 (C–COOH), 126.3 (=CHTe‐Cl_4_), 129.2 (=CN^+^), 134.6 (CH_pyrazol_), 139.5 (CH_pyrazol_), 160.2 (COOH); elemental analysis calcd (%) for C_8_H_8_Cl_4_N_2_O_2_Te: C 22.16, H 1.86, Cl 32.71, N 6.46; found: C 22.19, H 1.83, Cl 32.68, N 6.51.

(3 E)‐6‐carboxy‐3‐[(tetrabromo‐λ5‐tellanyl) methylene]‐2,3‐dihydro‐1H‐pyrazolo [1,2‐a] pyrazol‐4‐ium 14. Yield 34 %. m.p. 211–212 °C ^1^H NMR (400 MHz, [D^6^] DMSO): δ=4.25 (t, J=6 Hz, 2H, CH_2_), 4.64 (t, J=7 Hz, 2H, NCH_2_), 8.22 (s, 1H, =CHTe), 9.14 (s, 1H, CH_pyrazol_), 10.01 (s, 1H, CH_pyrazol_), 13.64 (s, 1H, COOH). ^13^C NMR (101 MHz, DMSO D^6^): δ=30.1 (CH_2_), 48.3 (NCH_2_), 117.7 (C–COOH), 122.0 (=CHTe‐Br_4_), 129.2 (=CN^+^), 134.6 (CH_pyrazol_), 138.9 (CH_pyrazol_), 160.9 (COOH); elemental analysis calcd (%) for C8H8Br4 N2O2Te: C 15.72, H 1.32, Br 52.28, N 4.58; found: C 15.77, H 1.28, Br 52.31, N 4.54.

6‐(methoxycarbonyl)‐3‐[(tetrachloro‐λ5‐tellanyl) methyl]‐2,3‐dihydro‐1H‐pyrazolo [1,2‐a] pyrazol‐4‐ium 15. Yield 44 %. m.p. 206 °C ^1^H NMR (400 MHz, [D^6^] DMSO): δ=3.00 (m, 1H, CH_2_), 3.19 (m, 1H, CH_2_), 3.86 (s, 3H, OCH_3_), 4.06 (dd, J=6 Hz, J=6 Hz, 1H, NCH_2_), 4.29 (dd, J=5.6 Hz, J=5.2 Hz, 1H, NCH_2_), 4.60 (m, 1H, CH_2_Te), 4.67 (m, 1H, CH_2_Te), 5.80 (t, J=6 Hz, 1H, N^+^CH), 9.06 (s, 1H, CH_pyrazol_), 9.16 (s, 1H, CH_pyrazol_). ^13^C NMR (101 MHz, DMSO D^6^): δ=35.3 (CH_2_), 49.2 (NCH_2_), 53.1 (OCH_3_), 61.3 (CHN^+^), 63.9 (CH_2_Te‐Cl_4_), 119.2 (C–COOCH_3_), 133.1 (CH_pyrazol_), 133.4 (CH_pyrazol_), 160.6 (COOCH_3_); elemental analysis calcd (%) for C_9_H_12_Cl_4_N_2_O_2_Te: C 24.04, H 2.69, Cl 31.54, N 6.23; found: C 24.11, H 2.74, Cl 31.51, N 6.20.

6‐(methoxycarbonyl)‐3‐[(tetrabromo‐λ5‐tellanyl) methyl]‐2,3‐dihydro‐1H‐pyrazolo [1,2‐a] pyrazol‐4‐ium 16. Yield 45 %. m.p. 193 °C ^1^H NMR (400 MHz, [D^6^] DMSO): δ=3.05 (m, 1H, CH_2_), 3.25 (m, 1H, CH_2_), 3.86 (s, 3H, OCH_3_), 4.26 (dd, J=7.2 Hz, J=6.8 Hz, 1H, NCH_2_), 4.46 (dd, J=4 Hz, J=4.4 Hz, 1H, NCH_2_), 4.61 (m, 1H, CH_2_Te), 4.68 (m, 1H, CH_2_Te), 5.86 (t, J=5.6 Hz, 1H, N^+^CH), 9.07 (s, 1H, CH_pyrazol_), 9.18 (s, 1H, CH_pyrazol_). ^13^C NMR (101 MHz, DMSO D^6^): δ=33.1 (CH_2_), 49.0 (NCH_2_), 52.6 (OCH_3_), 56.2 (CH_2_Te‐Br_4_), 61.7 (CHN^+^), 119.3 (C–COOCH_3_), 132.3 (CH_pyrazol_), 133.2 (CH_pyrazol_), 160.0 (COOCH_3_); elemental analysis calcd (%) for C_9_H_12_Br_4_N_2_O_2_Te: C 17.23, H 1.93, Br 50.94, N 4.46; found: C 17.30, H 1.96, Br 50.90, N 4.41.

6‐carboxy‐3‐[(tetrachloro‐λ5‐tellanyl) methyl]‐2,3‐dihydro‐1H‐pyrazolo [1,2‐a] pyrazol‐4‐ium 17. Yield 23 %. m.p. 206–207 °C ^1^H NMR (400 MHz, [D^6^] DMSO): δ=2.98 (m, 1H, CH_2_), 3.18 (m, 1H, CH_2_), 4.06 (dd, J=6 Hz, J=5.6 Hz, 1H, NCH_2_), 4.29 (dd, J=5.6 Hz, J=6 Hz, 1H, NCH_2_), 4.58 (m, 1H, CH_2_Te), 4.66 (m, 1H, CH_2_Te), 5.81 (m, 1H, N^+^CH) 8.96 (s, 1H, CH_pyrazol_), 9.05 (s, 1H, CH_pyrazol_), 13.19 (s, 1H, COOH). ^13^C NMR (101 MHz, DMSO D^6^): δ=35.4 (CH_2_), 49.1 (NCH_2_), 61.1 (CHN^+^), 64.1 (CH_2_Te‐Cl_4_), 120.7 (C–COOCH_3_), 133.1 (CH_pyrazol_), 133.4 (CH_pyrazol_), 161.6 (COOH); elemental analysis calcd (%) for C_8_H_8_C_l4_N_2_O_2_Te: C 22.16, H 1.86, Cl 32.71, N 6.46; found: C 22.19, H 1.83, Cl 32.68, N 6.51.

### X‐Ray Experimental Part

The colourless crystals of compound 10 (C9H10Cl4 N2O2Te) are triclinic. At 173 K a=7.9781 (3), b=10.0132 (4), c=11.0541 (4) Å, α=66.175 (2)°, β=69.073 (2)°, γ=71.620 (2)°, V=739.26 (5) Å3, Mr=447.59, Z=2, space group P, dcalc=2.011 g/cm3, *(MoK*)=2.729 mm^−1^, F(000)=428. Intensities of 13945 reflections (4301 independent, Rint=0.032) were measured on the Bruker APEX II diffractometer (graphite monochromated MoKα radiation, CCD detector, ϕ‐ and ω‐scaning, 2Θmax=50°). The structure was solved by direct method using OLEX2^[40]^ package with SHELXT^[41]^ and SHELXL modules.^[42]^ The absorption correction was done using ‘numerical’ method (Tmin=0.3285, Tmax=0.8357). Positions of the hydrogen atoms were located from electron density difference maps and refined using “riding” model with Uiso=nUeq (n=1.5 for hydroxyl group and n=1.2 for other hydrogen atoms) of the carrier atom. Full‐matrix least‐squares refinement against F2 in anisotropic approximation for non‐hydrogen atoms using 4301 reflections was converged to wR2=0.1012 (R1=0.0357 for 3514 reflections with F>4σ(F), S=1.080). The final atomic coordinates, and crystallographic data for molecule 10 have been deposited to with the Cambridge Crystallographic Data Centre, 12 Union Road, CB2 1EZ, UK (fax: +44–1223–336033; e‐mail: deposit@ccdc.cam.ac.uk) and are available on request quoting the deposition numbers CCDC 2403861).

The colourless crystals of compound 11 (C_9_H_10_Br_4_N_2_O_2_Te) are triclinic. At 173 K a=8.2563 (3), b=10.4003 (5), c=11.1334 (5) Å, α=64.870 (2)°, β=69.531 (2)°, γ=70.258 (2)°, V=790.42 (6) Å3, Mr=625.43, Z=2, space group P, dcalc=2.628 g/cm^3^, *(MoK*)=11.987 mm^−1^, F(000)=572. Intensities of 14139 reflections (4624 independent, Rint=0.050) were measured on the Bruker APEX II diffractometer (graphite monochromated MoKα radiation, CCD detector, ϕ‐ and ω‐scaning, 2Θmax=50°). The structure was solved by direct method using OLEX2^[40]^ package with SHELXT^[41]^ and SHELXL modules.^[42]^ The absorption correction was done using ‘numerical’ method (T min=0.0394, T max=0.2080). Positions of the hydrogen atoms were located from electron density difference maps and refined using “riding” model with Uiso=nUeq (n=1.5 for hydroxyl group and n=1.2 for other hydrogen atoms) of the carrier atom. Full‐matrix least‐squares refinement against F2 in anisotropic approximation for non‐hydrogen atoms using 4624 reflections was converged to wR2=0.0904 (R1=0.0397 for 3086 reflections with F>4σ(F), S=1.009). Deposition Numbers CCDC 2403861 (for structure 10) and 2403862 (for structure 11) contain the supplementary crystallographic data for this paper. These data are provided free of charge by the joint Cambridge Crystallographic Data Centre and Fachinformationszentrum Karlsruhe Access Structures service http://www.ccdc.cam.ac.uk/structures.

## Conflict of Interests

The authors declare no conflict of interest.

## Supporting information

As a service to our authors and readers, this journal provides supporting information supplied by the authors. Such materials are peer reviewed and may be re‐organized for online delivery, but are not copy‐edited or typeset. Technical support issues arising from supporting information (other than missing files) should be addressed to the authors.

Supporting Information

## Data Availability

The data that support the findings of this study are available in the supplementary material of this article.
