# Supplementary material for: Unexpected Tellurohalogenation of Terminal N‐Alkynyl (Alkenyl) Derivatives of 4‐Functionalized Pyrazoles
Source: ChemistryOpen. 2025 Jan 26;14(8):e202400486. doi: 10.1002/open.202400486 (PMC12368882; doi:10.1002/open.202400486)
Supplement: Supplementary file 1 — Supporting Information [file OPEN-14-e202400486-s001.pdf]

ChemistryOpen

Supporting Information

Unexpected Tellurohalogenation of Terminal N-Alkynyl (Alkenyl) Derivatives of 4-Functionalized Pyrazoles

Marianna V. Povidaichyk, Svitlana V. Shishkina, Eugeniy M. Ostapchuk, and Mykhaylo Yu. Onysko*

Chemistry Open

Supporting Information

UNEXPECTED TELLUROHALOGENATION OF TERMINAL N-ALKYNYL(ALKENYL) DERIVATIVES OF 4-FUNCTIONALIZED PYRAZOLE

Marianna V. Povidaichyk, Svitlana V. Shishkina, Eugeniy M. Ostapchuk, Mykhaylo Yu.Onysko

Table of Contents

1. Reaction conditions for 1-pentynylpyrazole-4-carboxylic acid 6 cyclization…3
2. NMR Spectra……………………………………………………………………..4-31

Table S1. Interaction between 1-pentynylpyrazole-4-carboxylic acid **6** and tellurium tetrahalides at different conditions.

Entry	Halogen	Reaction time, *h*	Reaction temperature, ^0C	Ratio of reagents	Solvent	Yield, %
1	Cl	0.5	20	1:1:6	AcOH	traces
2	Cl	1	20	1:1:6	AcOH	traces
3	Cl	2	20	1:1:6	AcOH	15
4	Cl	6	20	1:1:6	AcOH	27
5	Cl	24	20	1:1:6	AcOH	41
6	Cl	48	20	1:1:6	AcOH	40
7	Cl	24	20	1:1:6	C_2H_5OH	8
8	Cl	24	20	1:1:6	$CHCl_3$	24
9	Cl	24	20	1:1:6	CH_2Cl_2	28
10	Cl	24	20	1:1:6	H_2O	tarring
11	Br	24	20	1:1:6	AcOH	38
12	Br	48	20	1:1:6	AcOH	38
13	Br	24	20	1:1:6	$CHCl_3$	28
14	Br	24	20	1:1:6	CH_2Cl_2	24
15	Br	24	20	1:1:6	H_2O	tarring

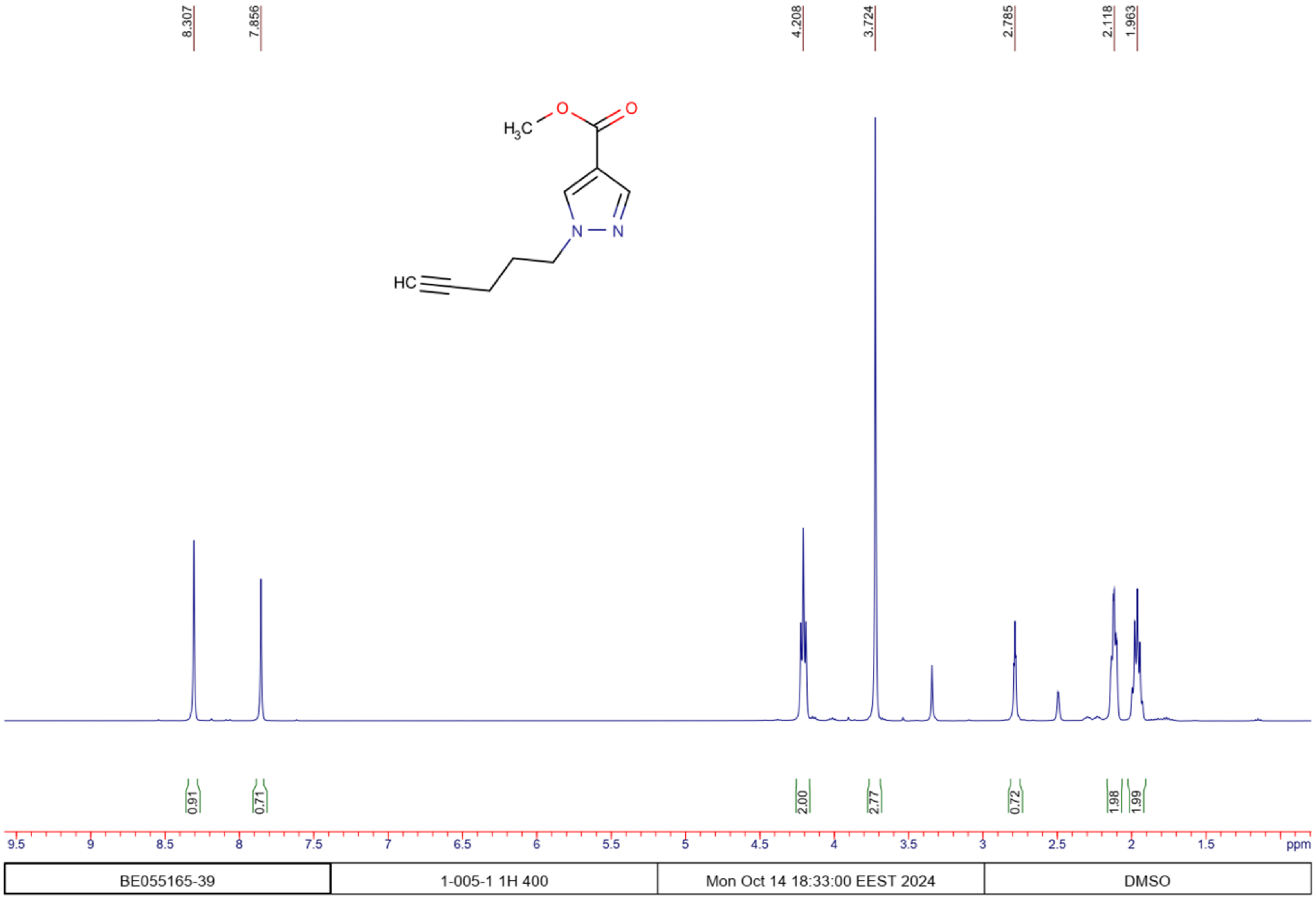

Figure S2. NMR ^{1}H compound 2

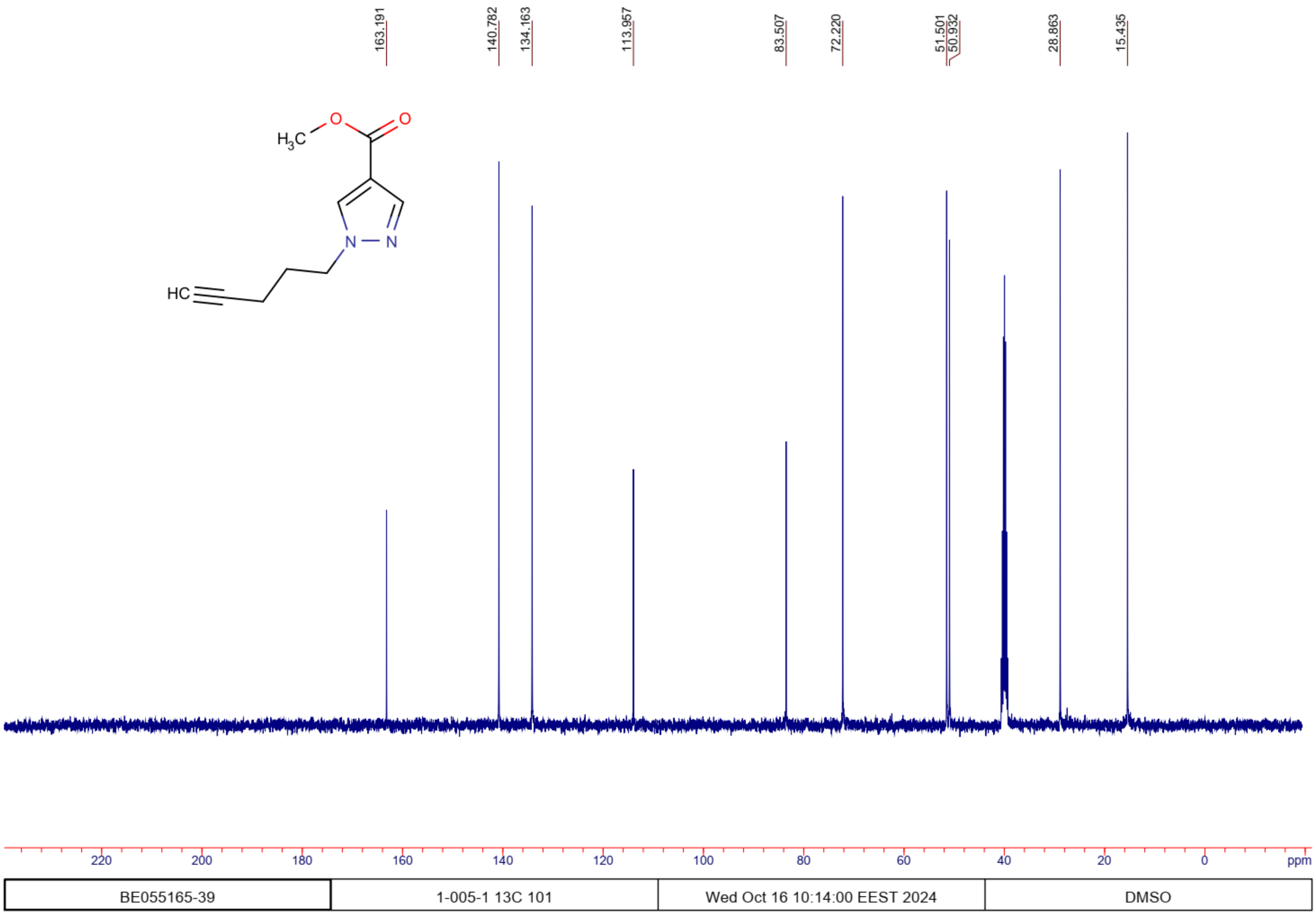

Figure S3. NMR ^{13}C compound 2

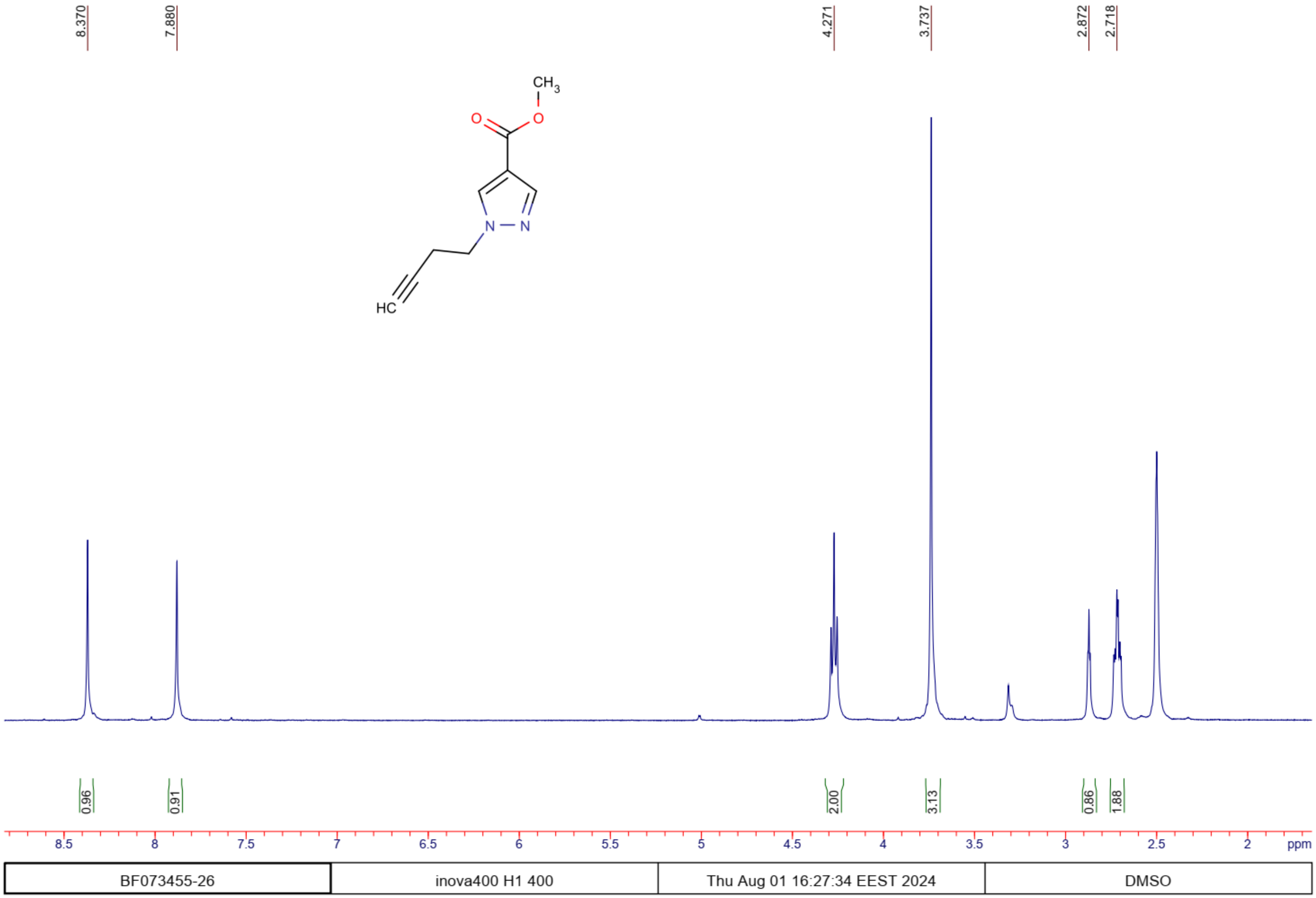

Figure S4. NMR ^{1}H compound 3

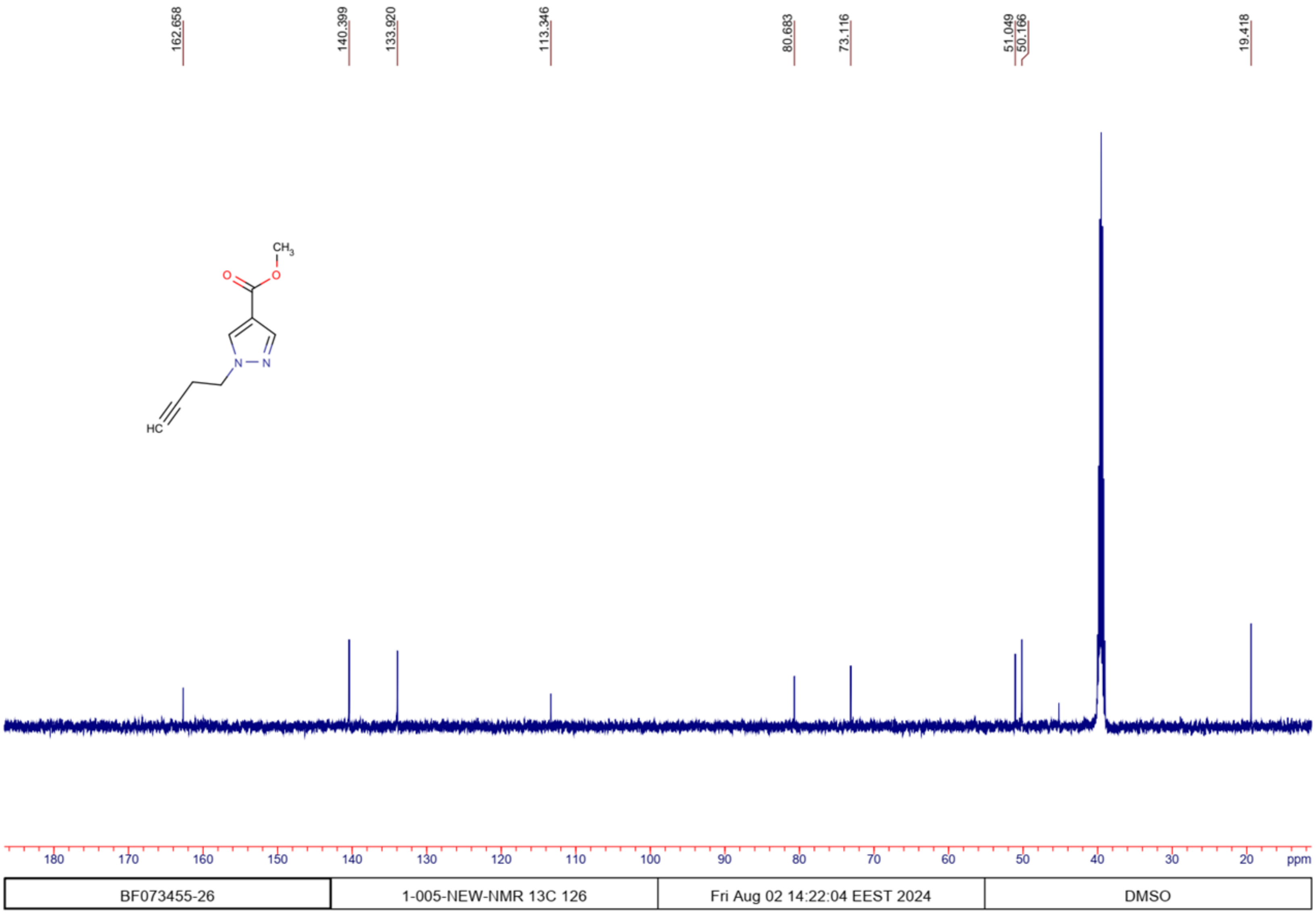

Figure S5. NMR 13C compound 3

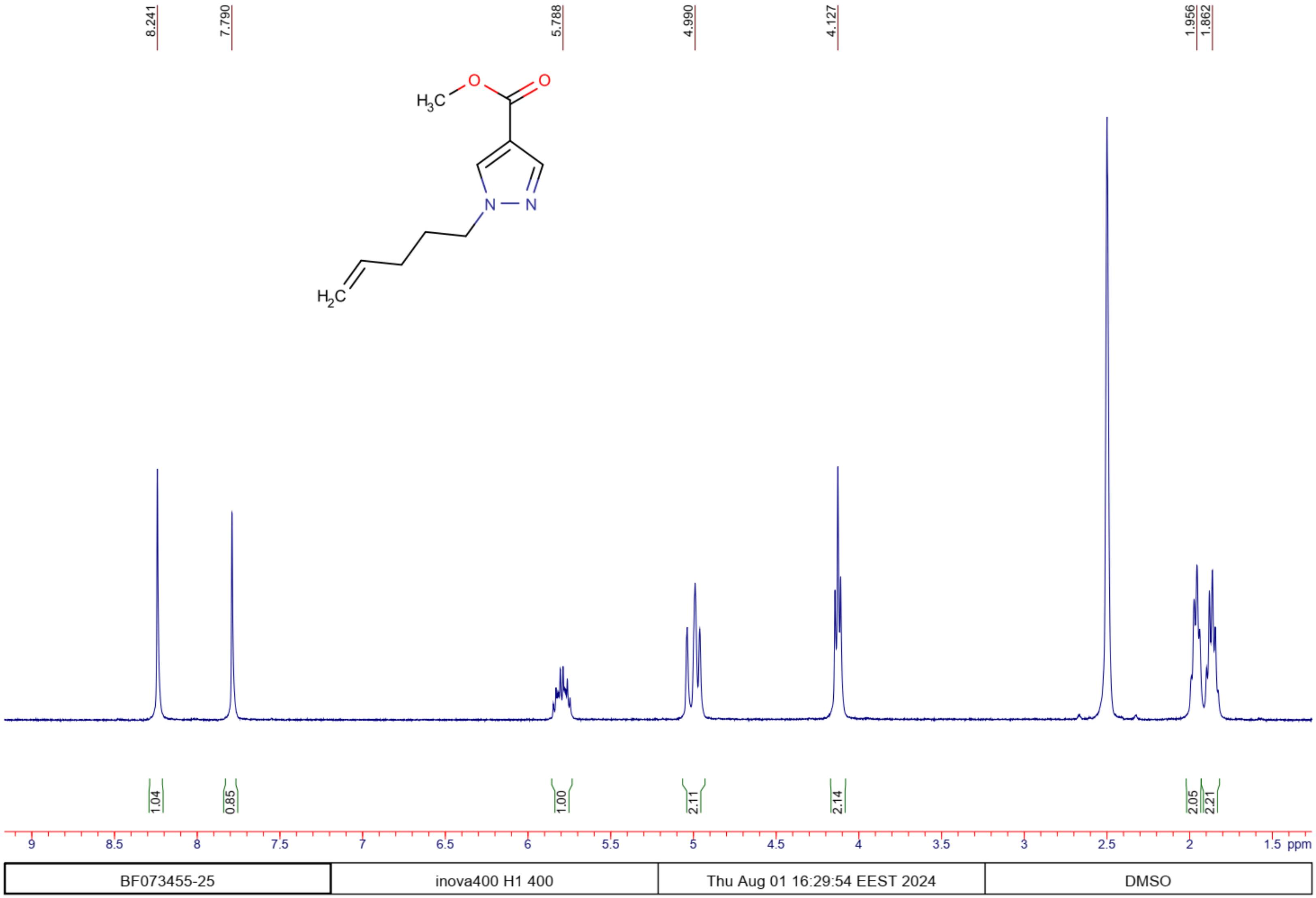

Figure S6. NMR ^{1}H compound 4

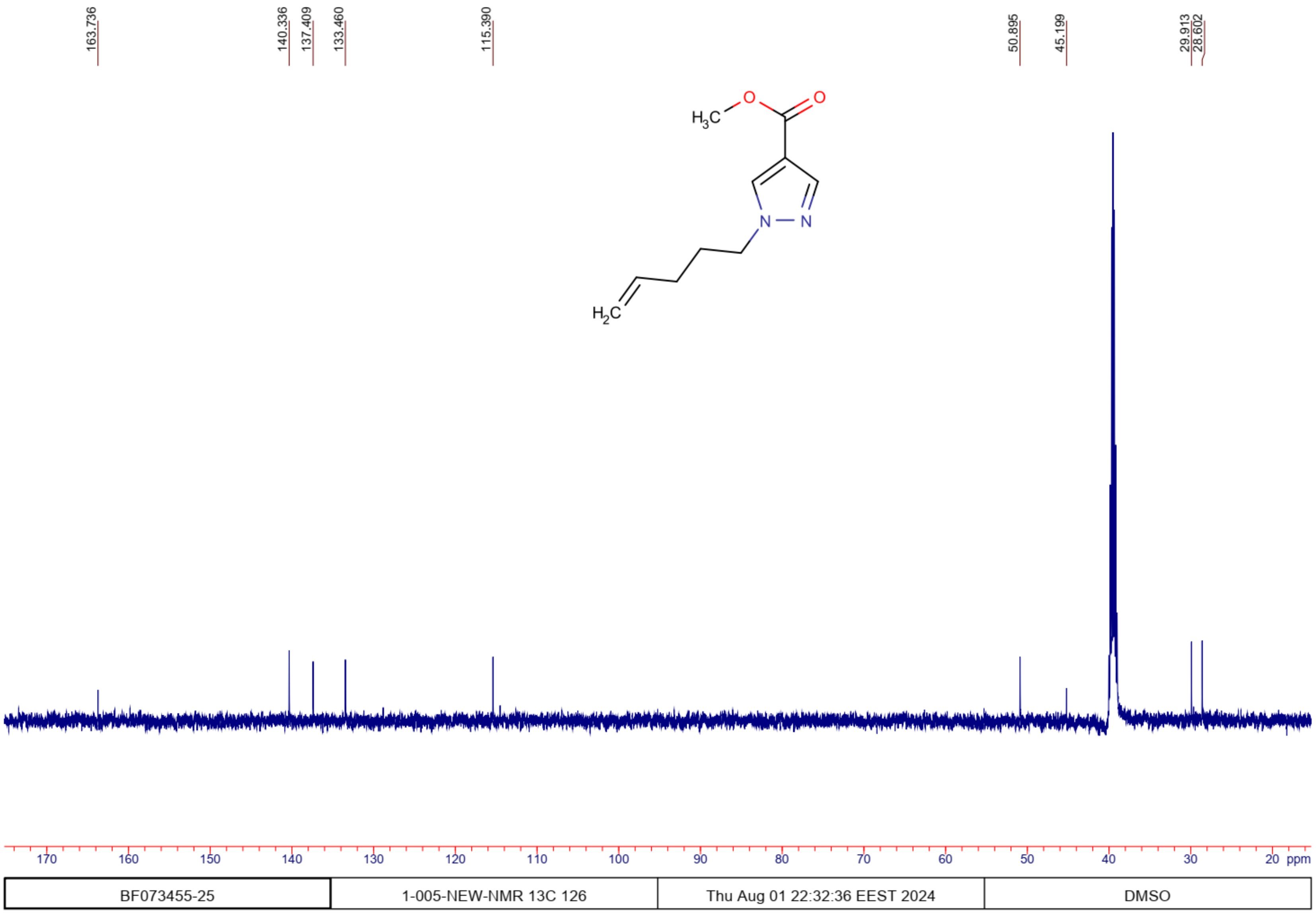

Figure S7. NMR ^{13}C compound 4

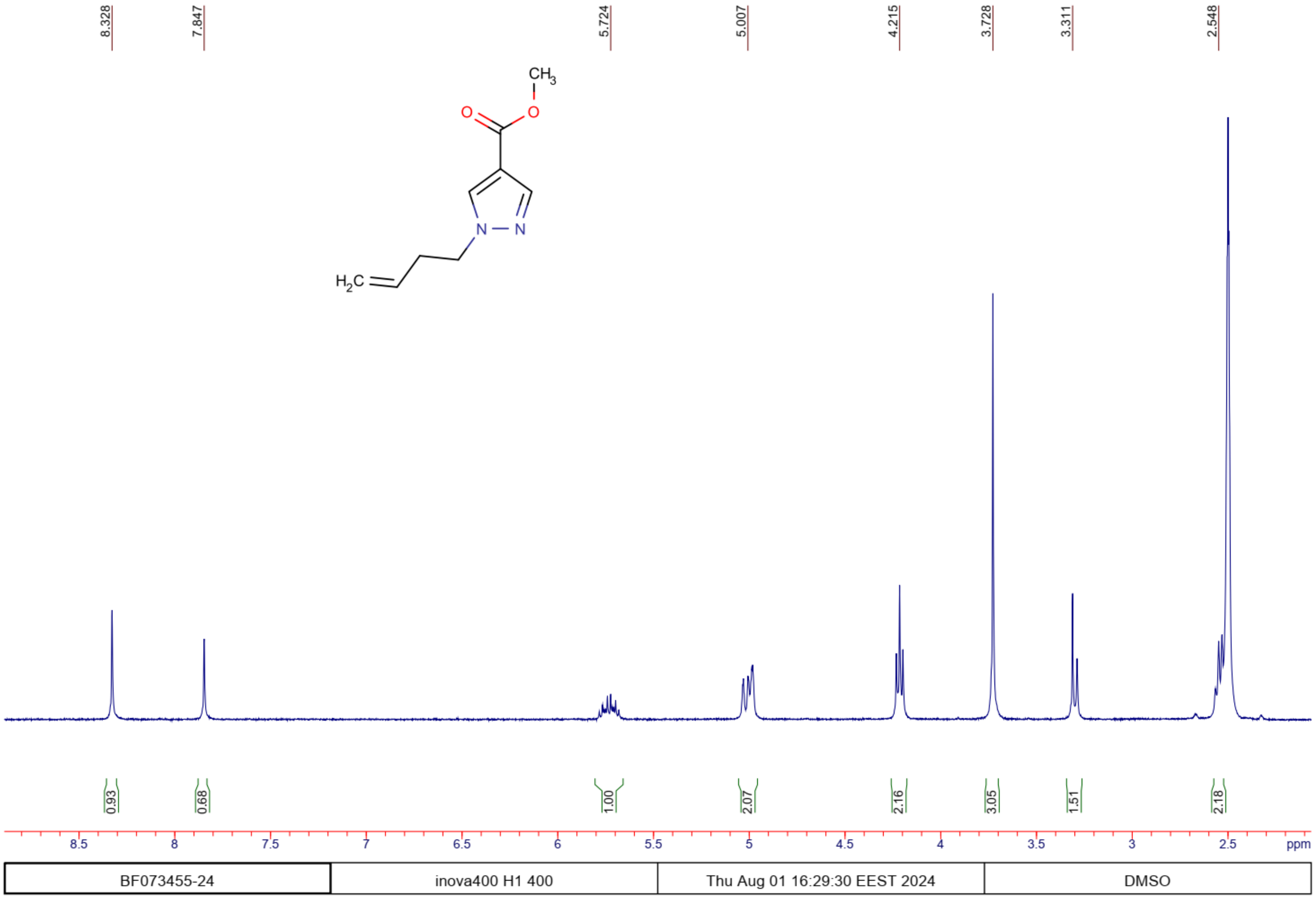

Figure S8. NMR ^{1}H compound 5

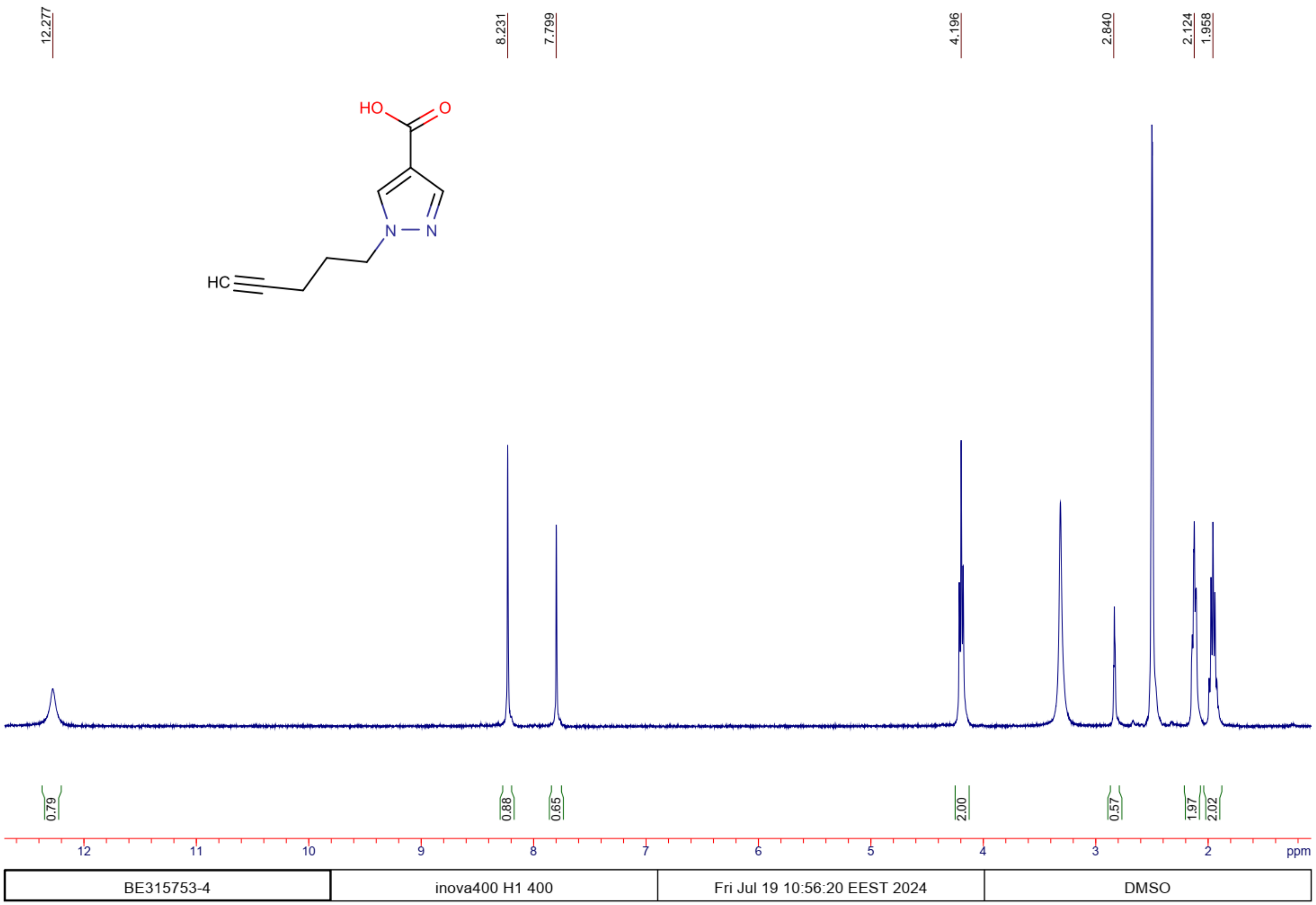

Figure S9. NMR ^{1}H compound 6

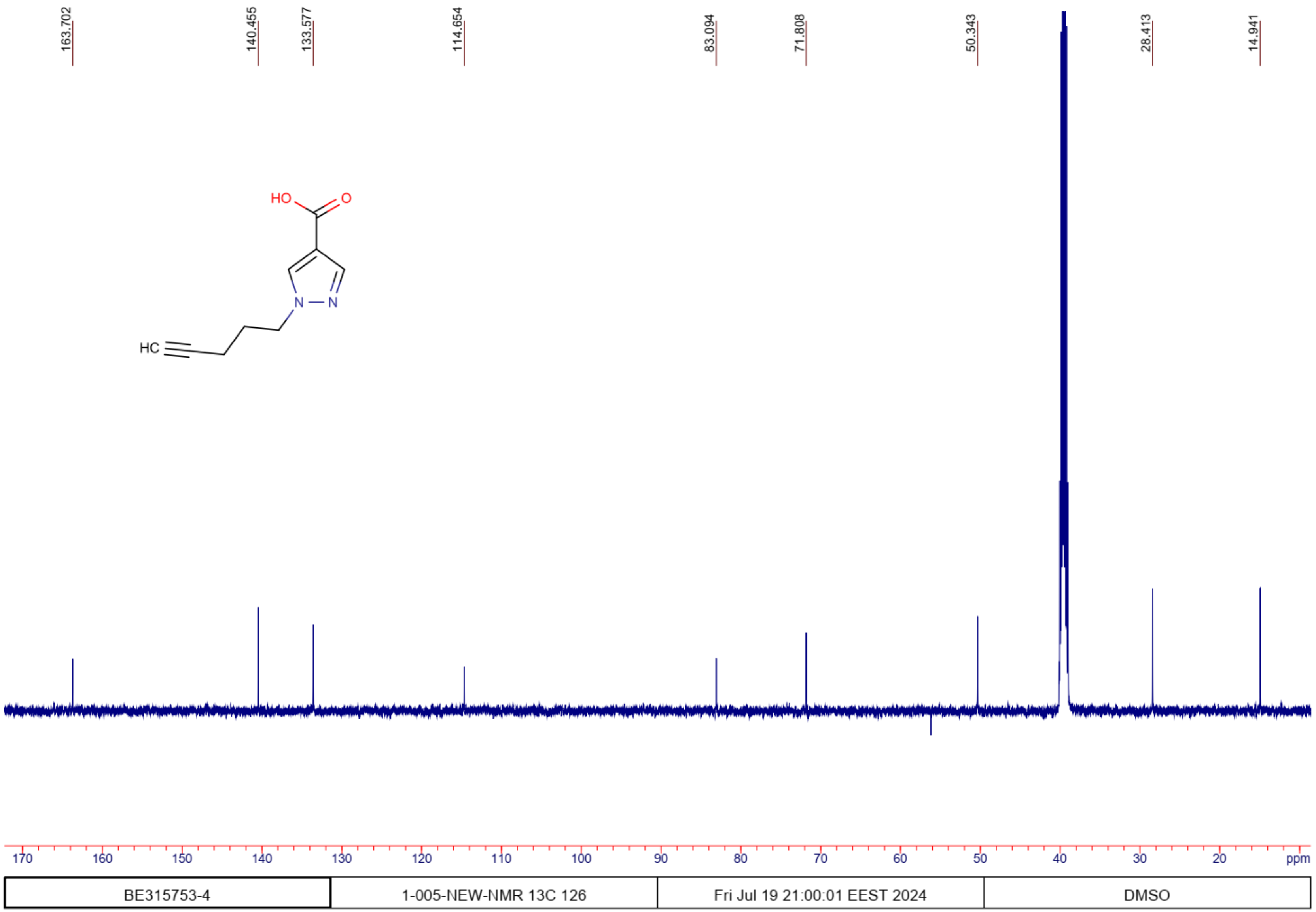

Figure S10. NMR ^{1}H compound 6

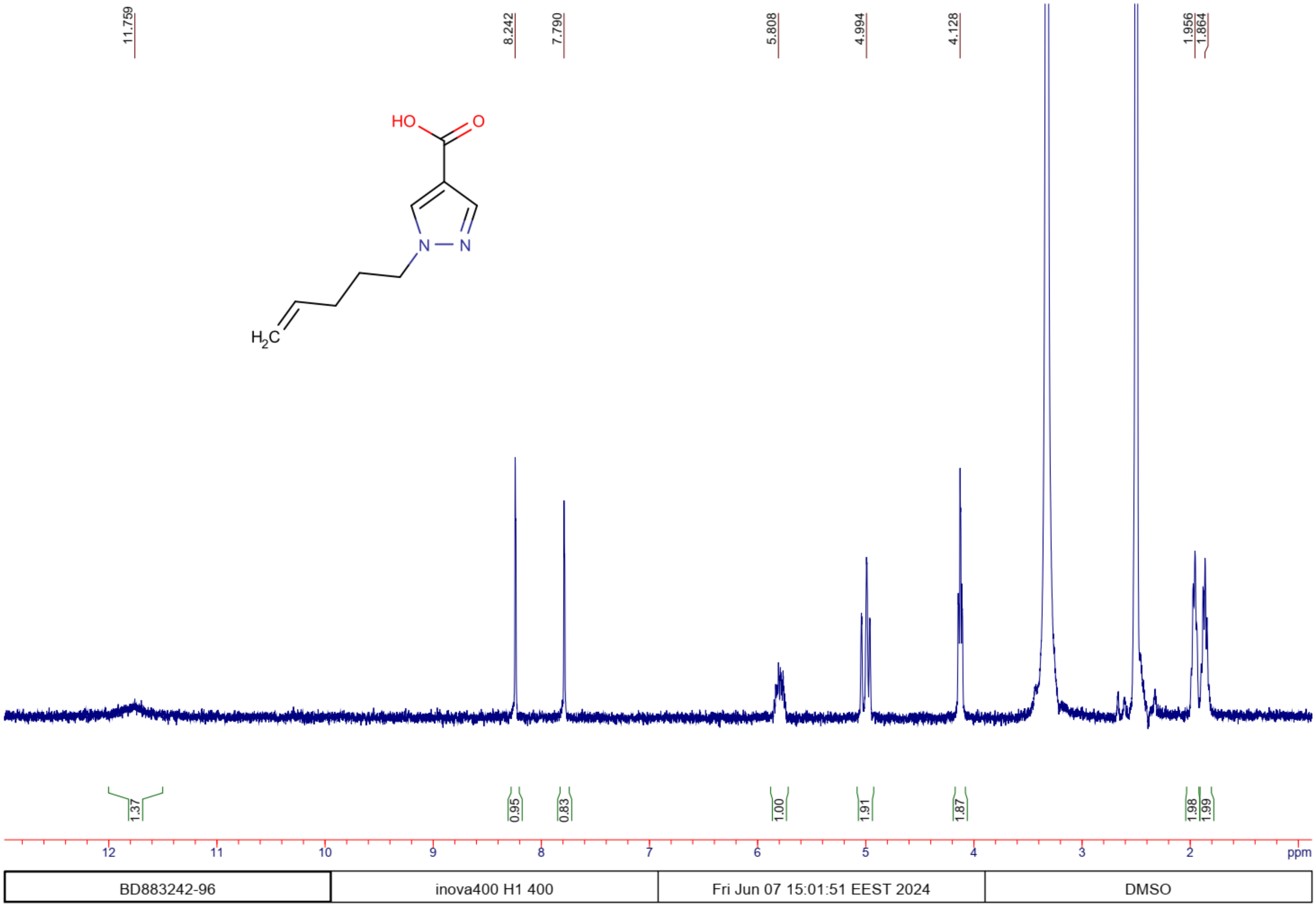

Figure S11. NMR ^{1}H compound 8

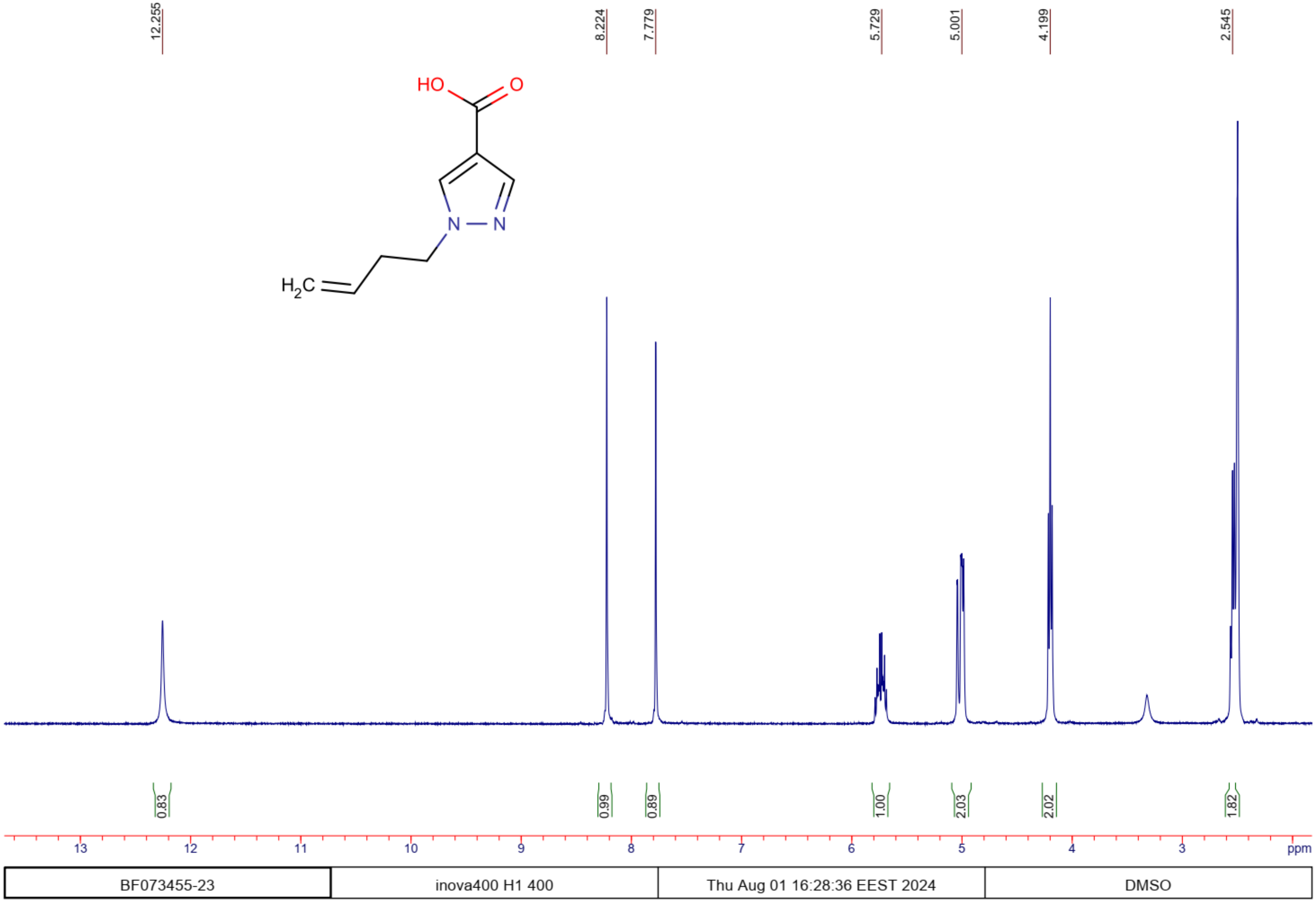

Figure S12. NMR ^{1}H compound 9

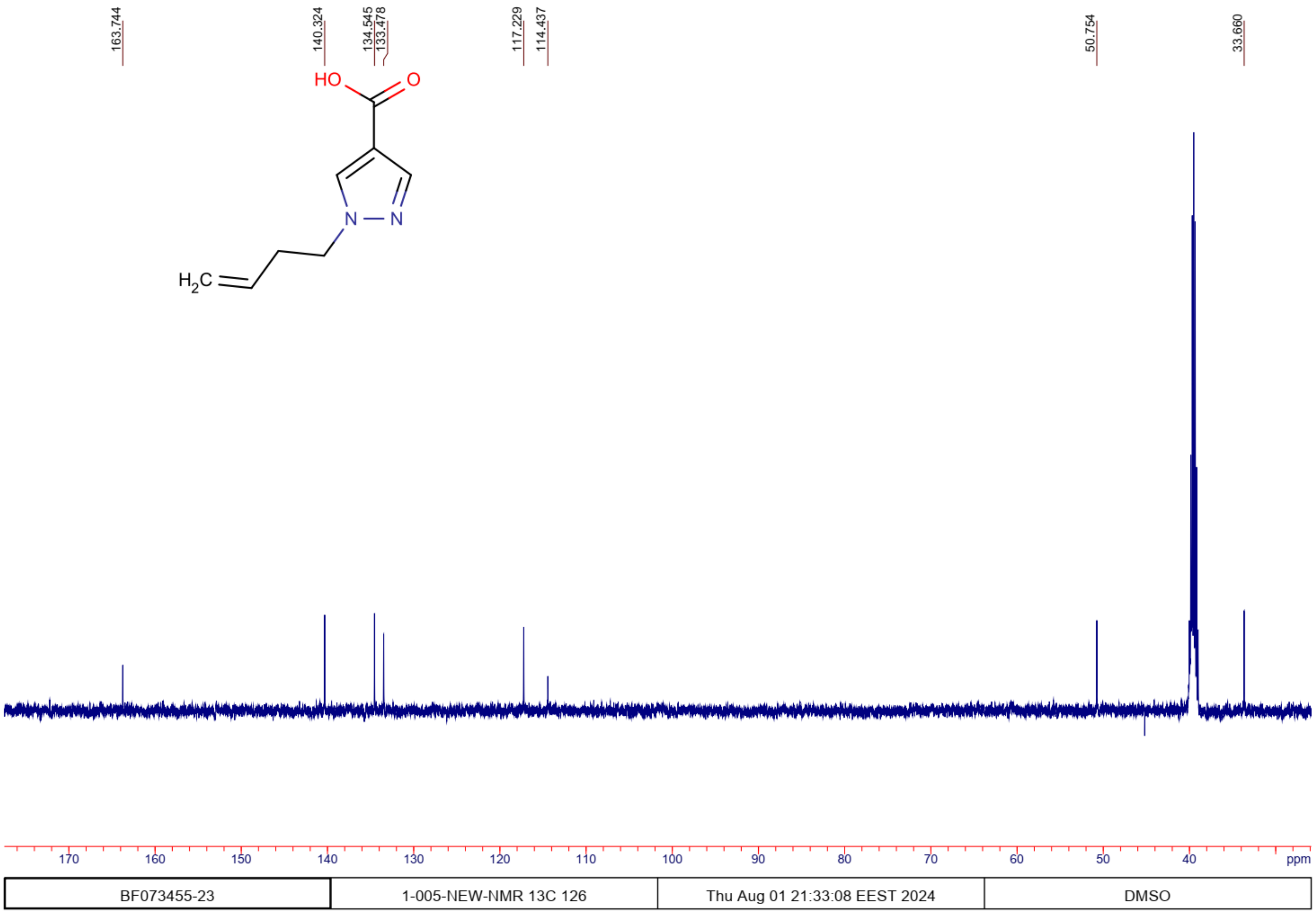

Figure S13. NMR ^{13}C compound 9

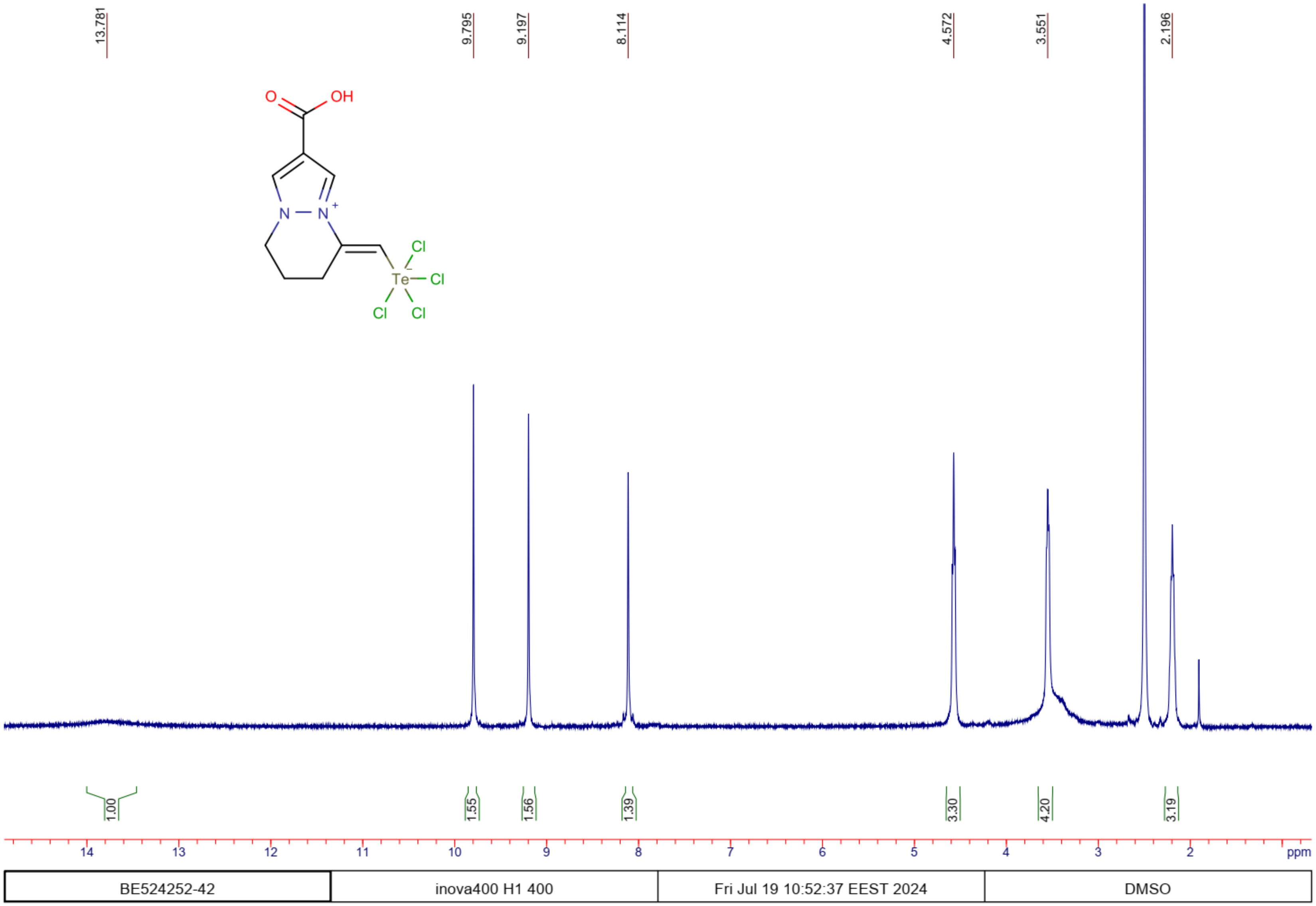

Figure S14. NMR ^{1}H compound 10

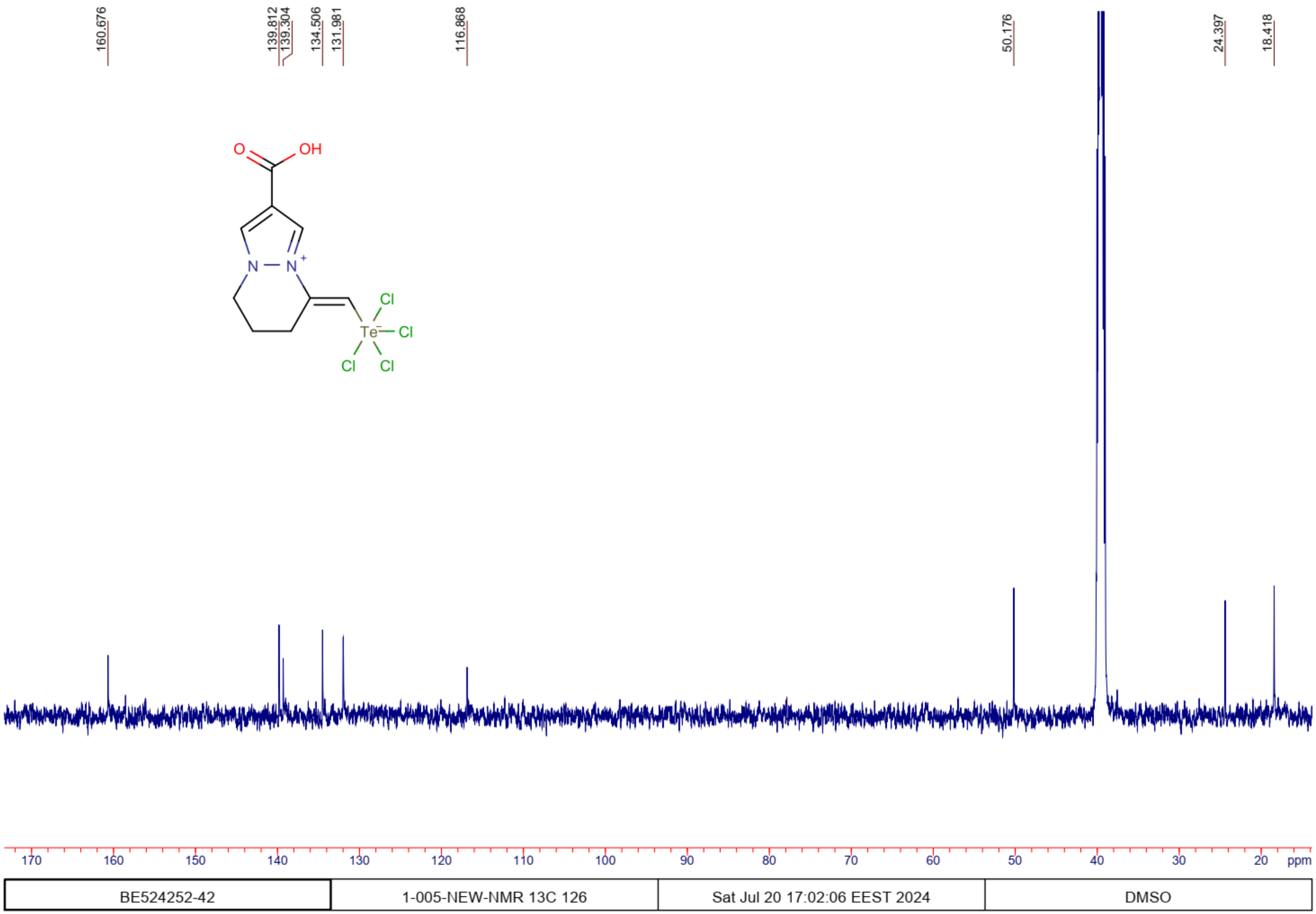

Figure S15. NMR ^{13}C compound 10

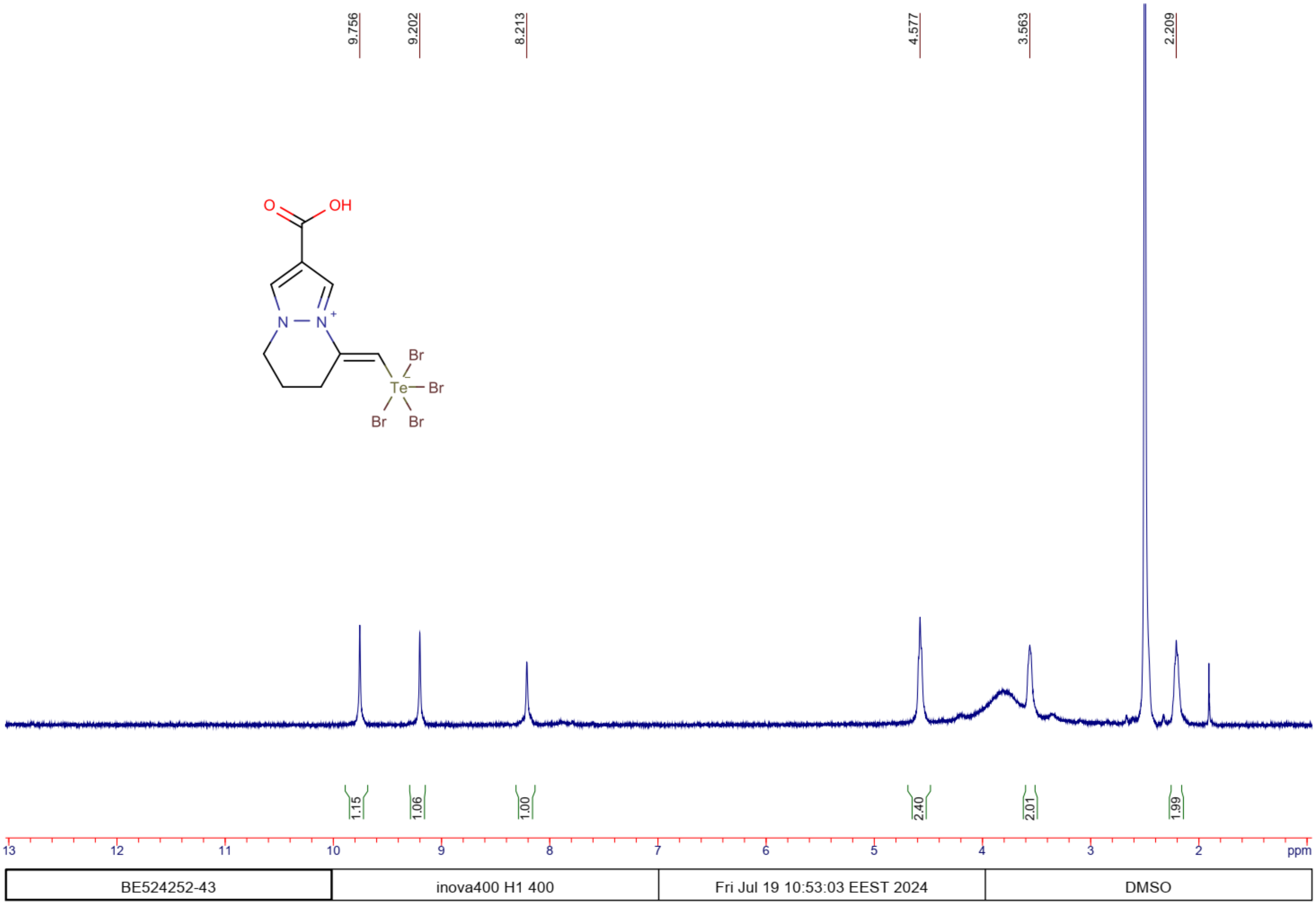

Figure S16. NMR ^{1}H compound 11

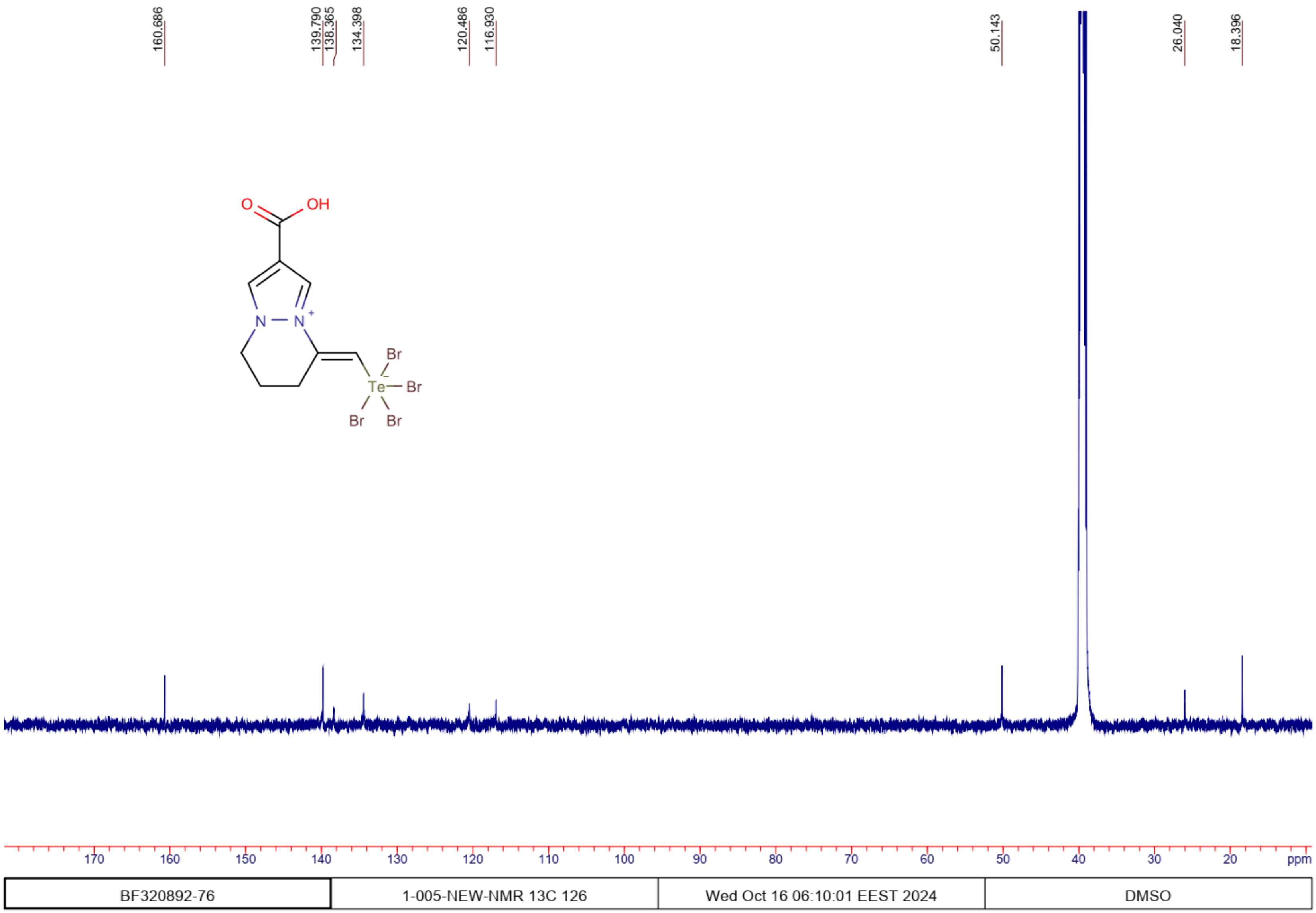

Figure S17. NMR ^{13}C compound 11

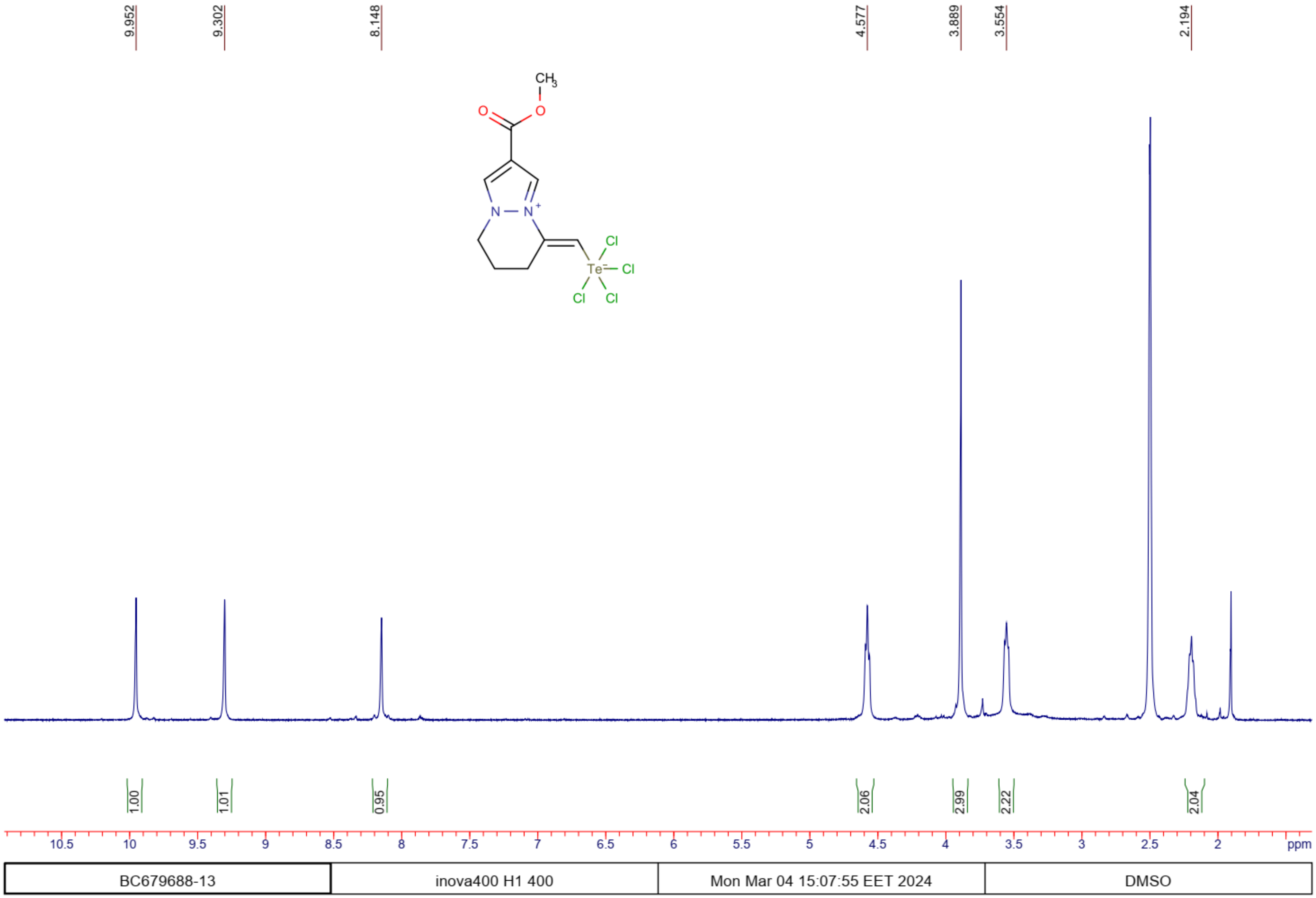

Figure S18. NMR ^{1}H compound 12

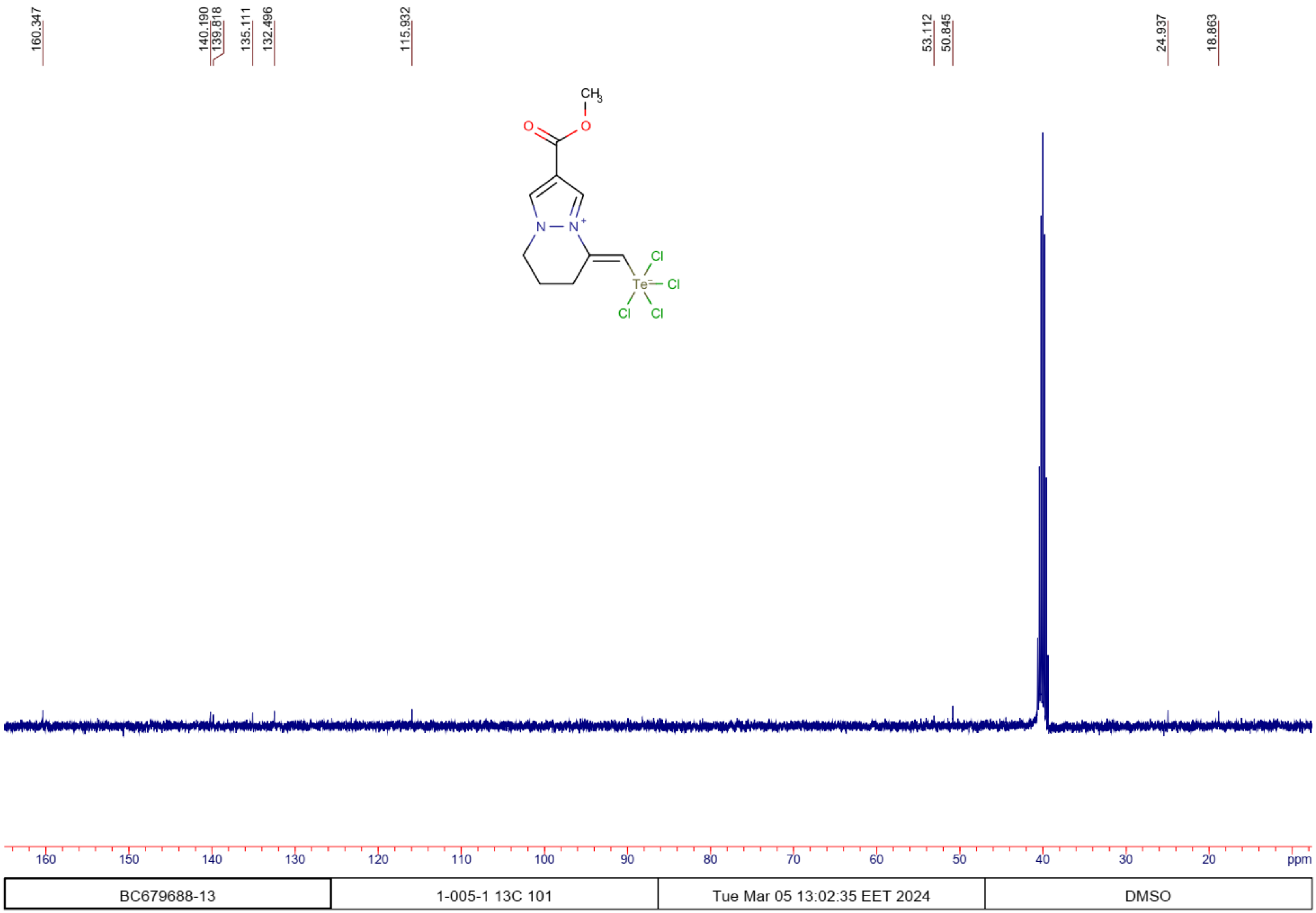

Figure S19. NMR ^{13}C compound 12

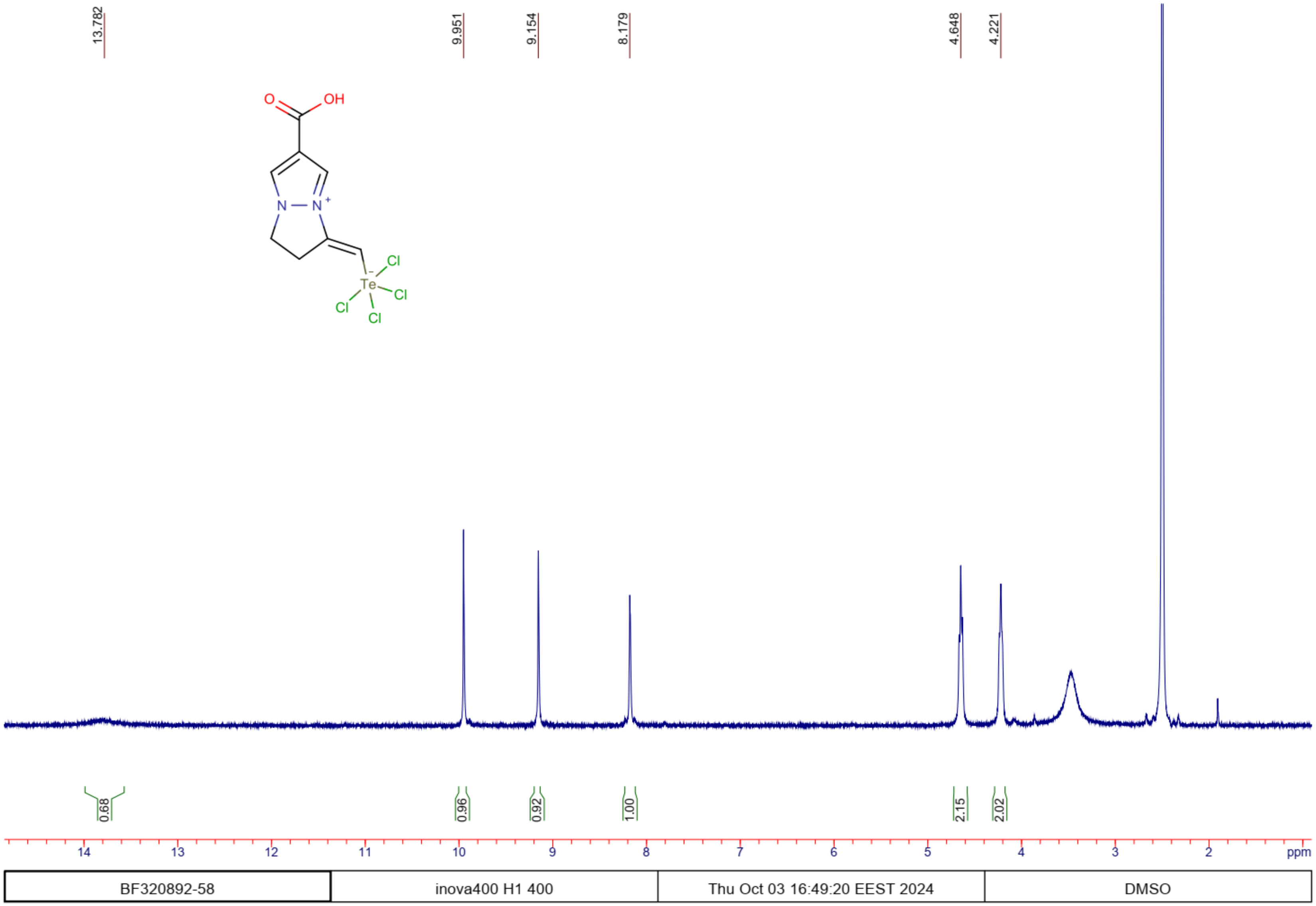

Figure S20. NMR ^{1}H compound 13

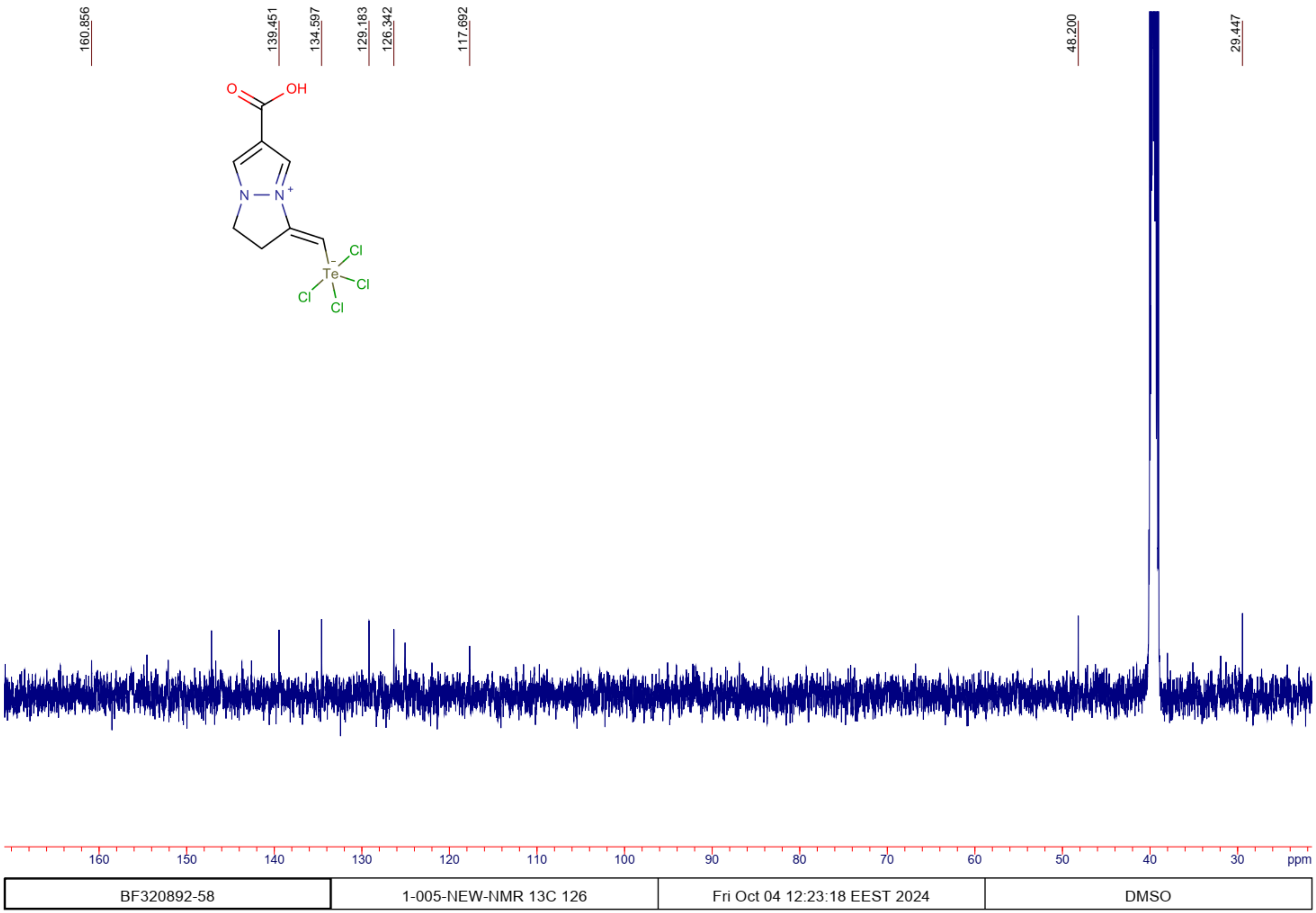

Figure S21. NMR ^{1}H compound 13

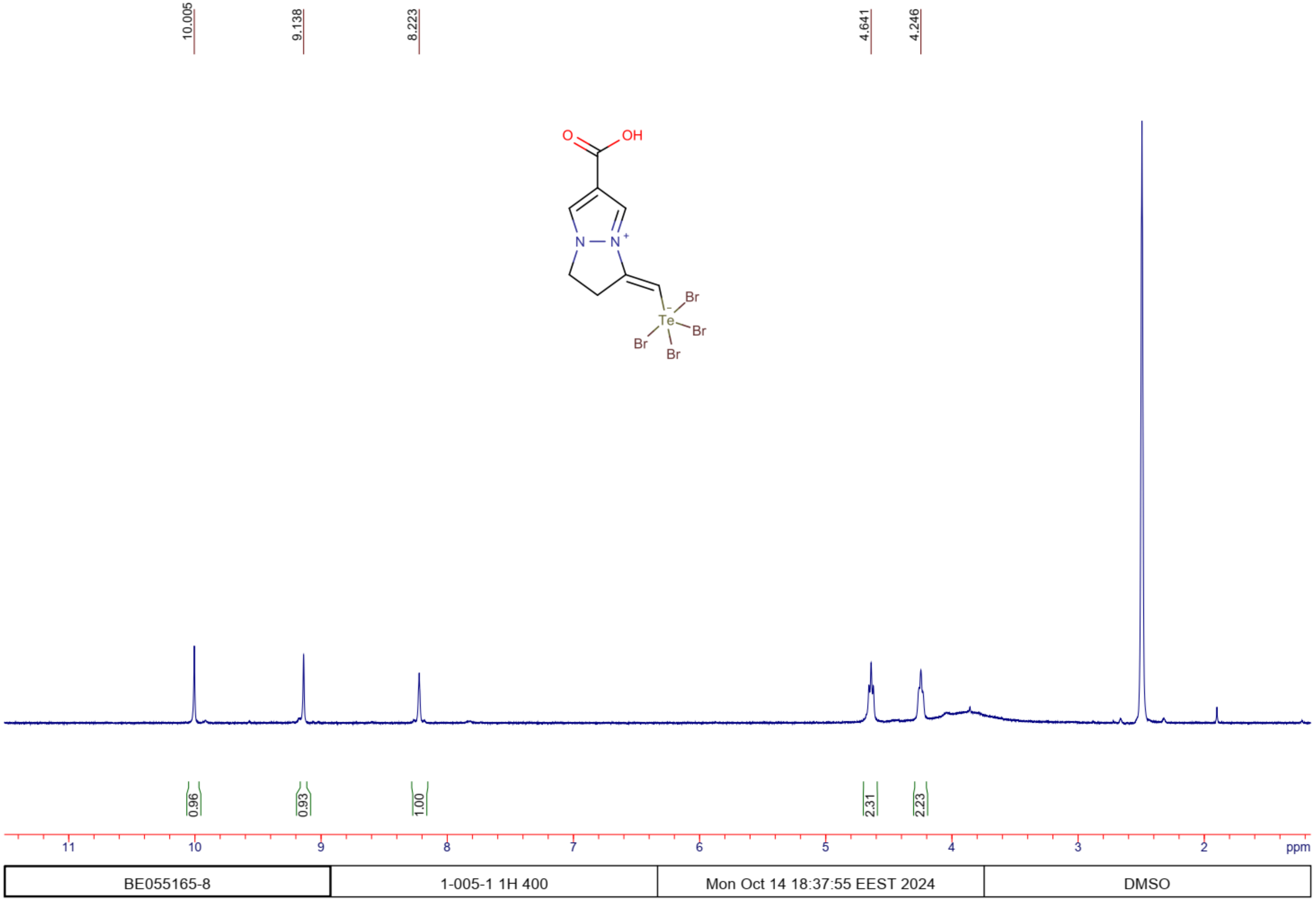

Figure S22. NMR ^{1}H compound 14

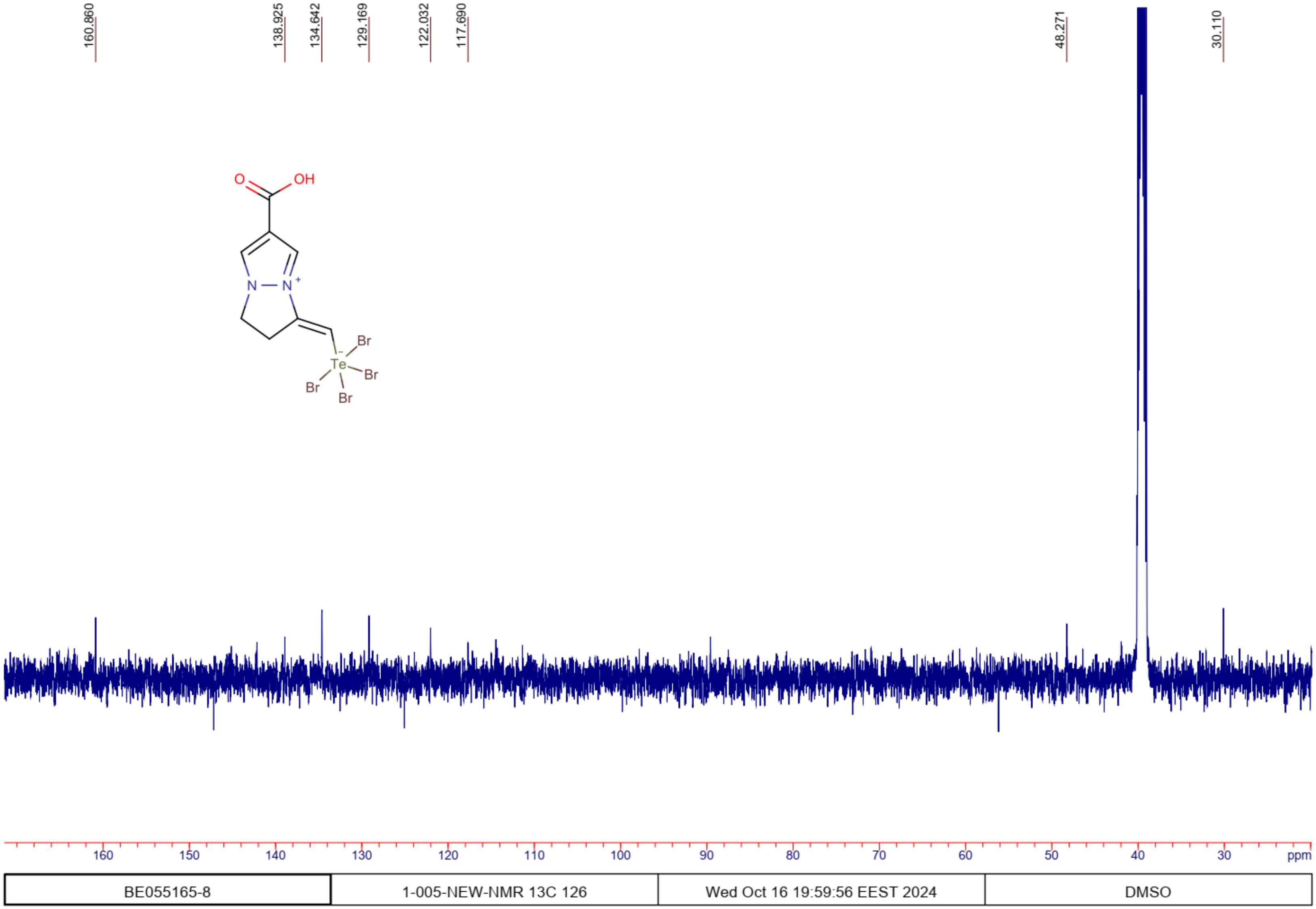

Figure S23. NMR ^{1}H compound 14

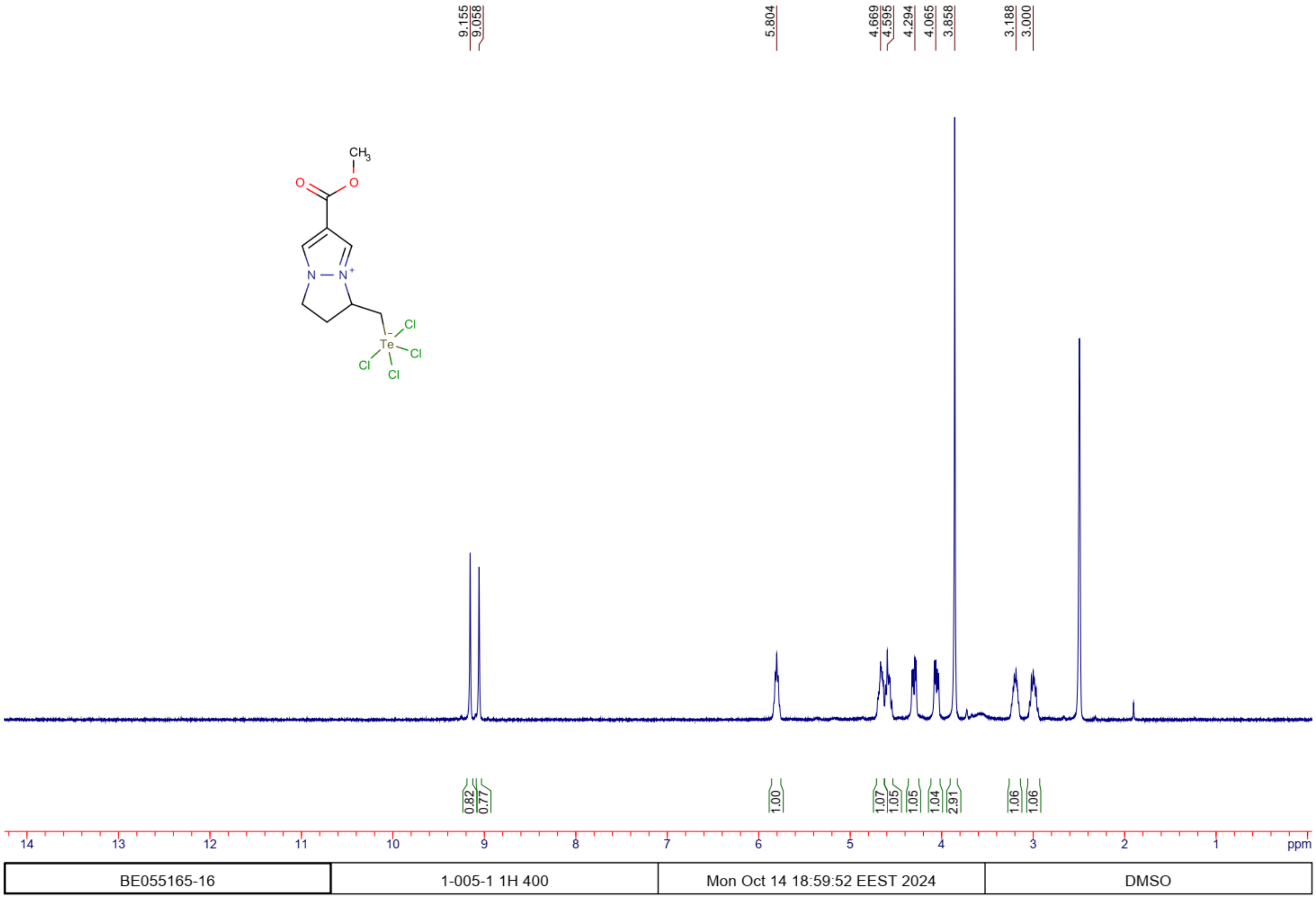

Figure S24. NMR ^{1}H compound 15

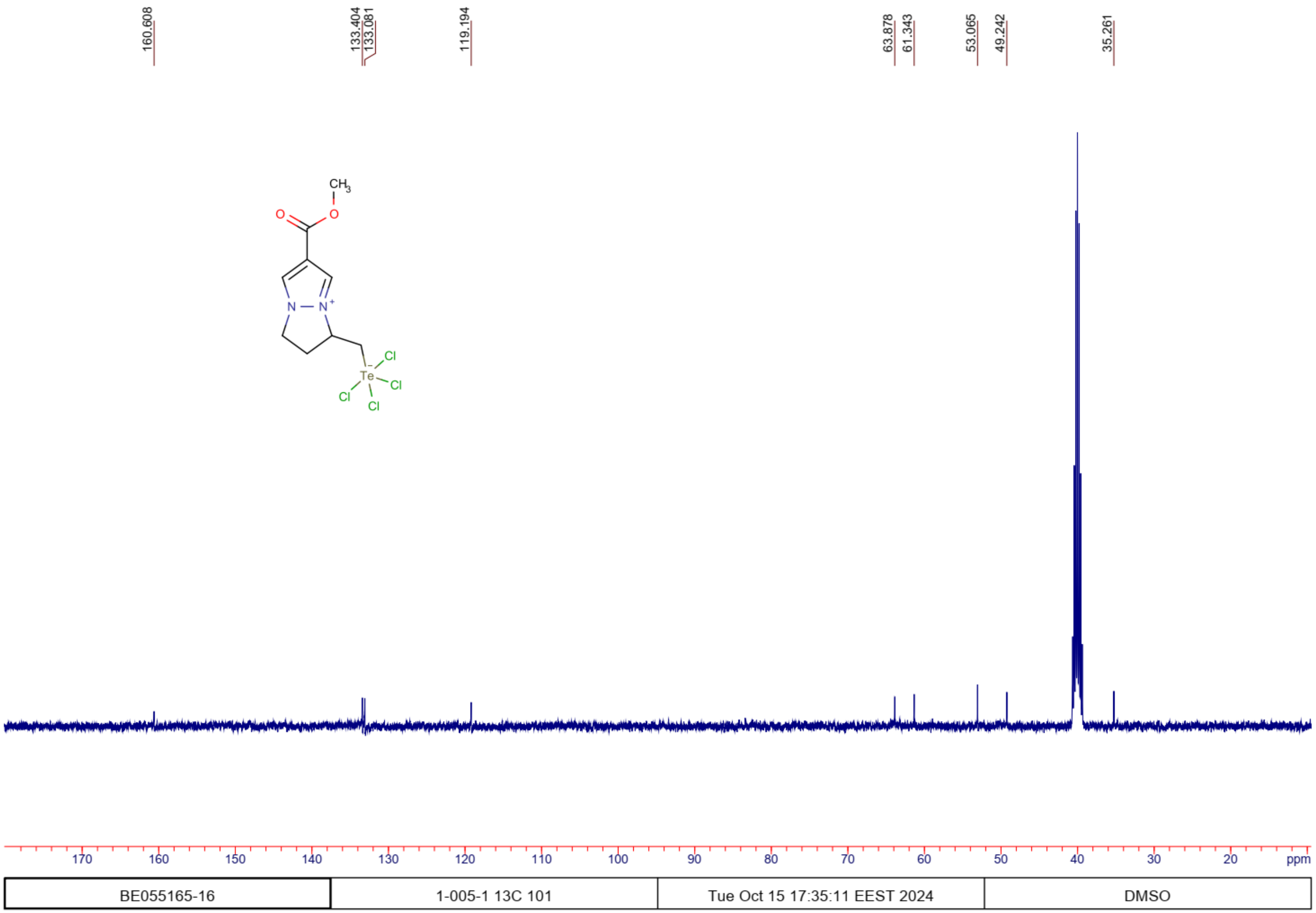

Figure S25. NMR ^{13}C compound 15

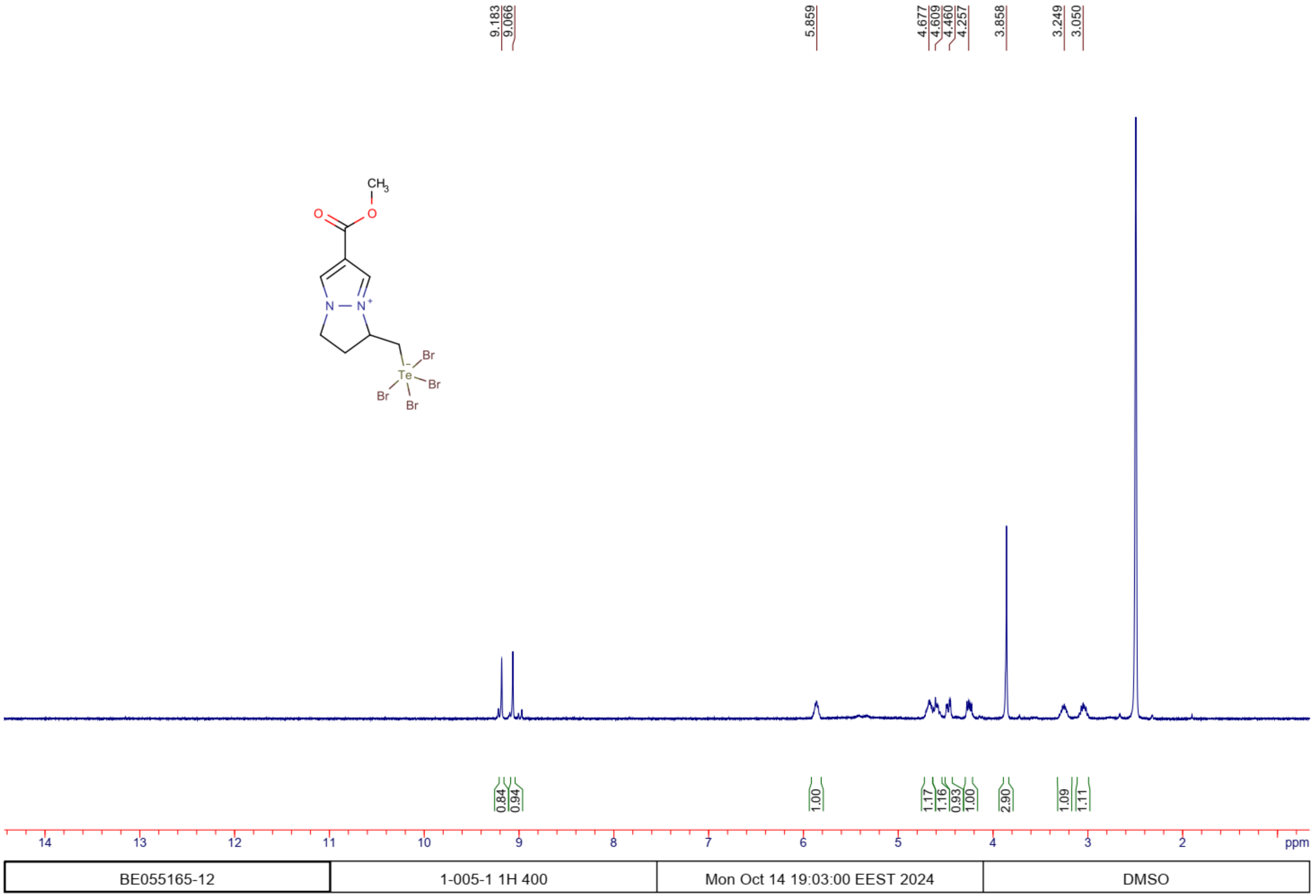

Figure S26. NMR ^{1}H compound 16

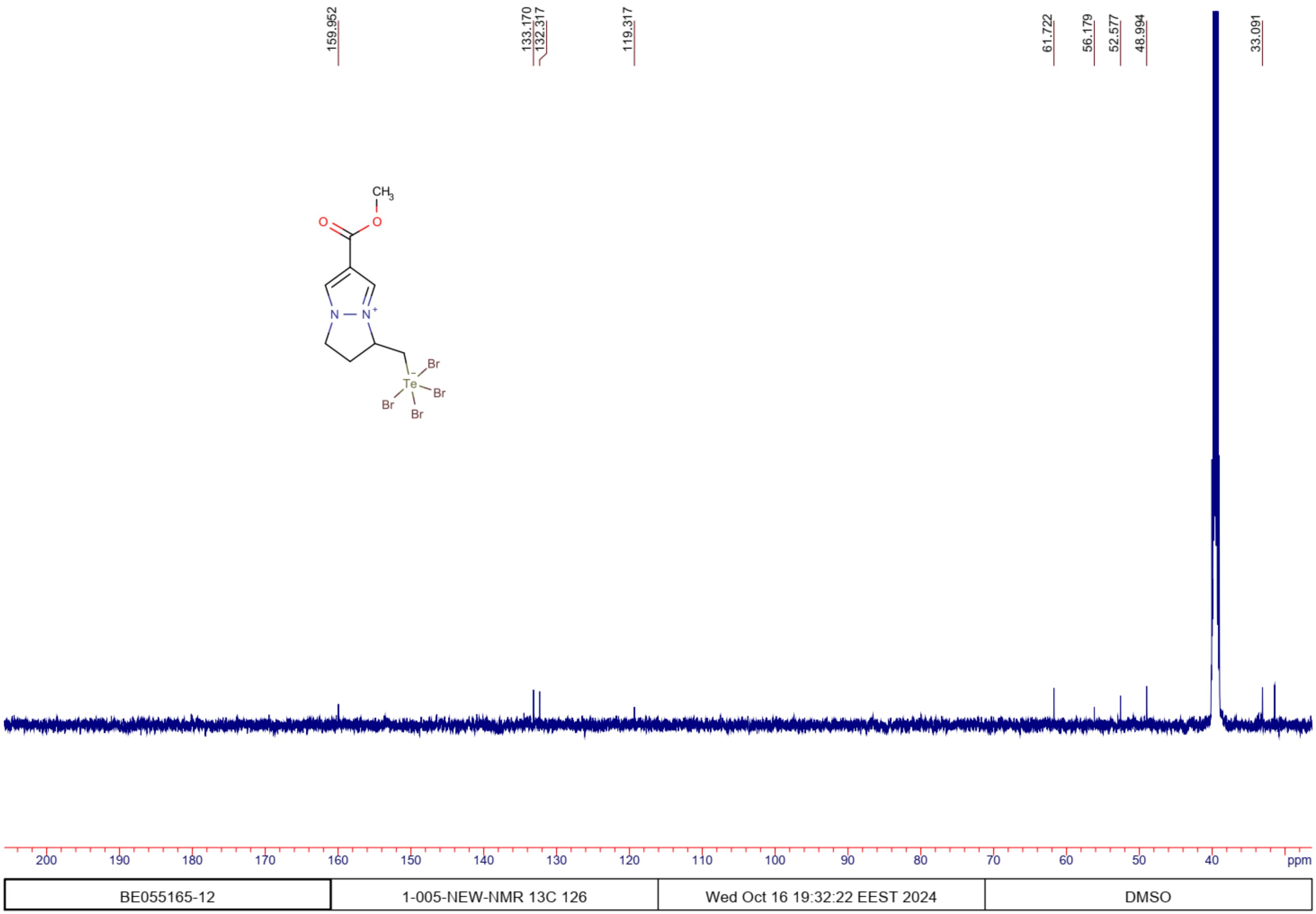

Figure S27. NMR ^{13}C compound 16

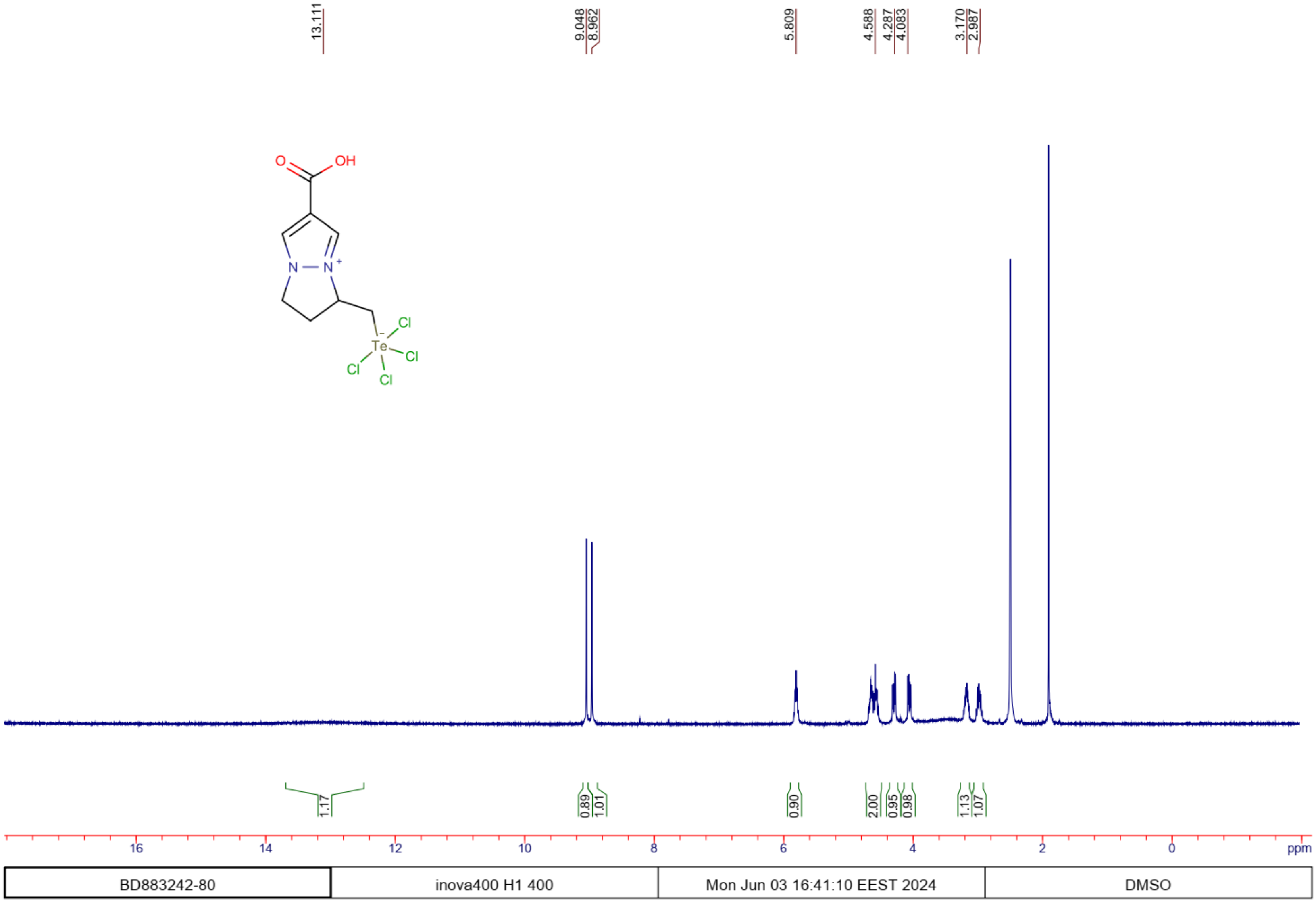

Figure S28. NMR ^{1}H compound 17

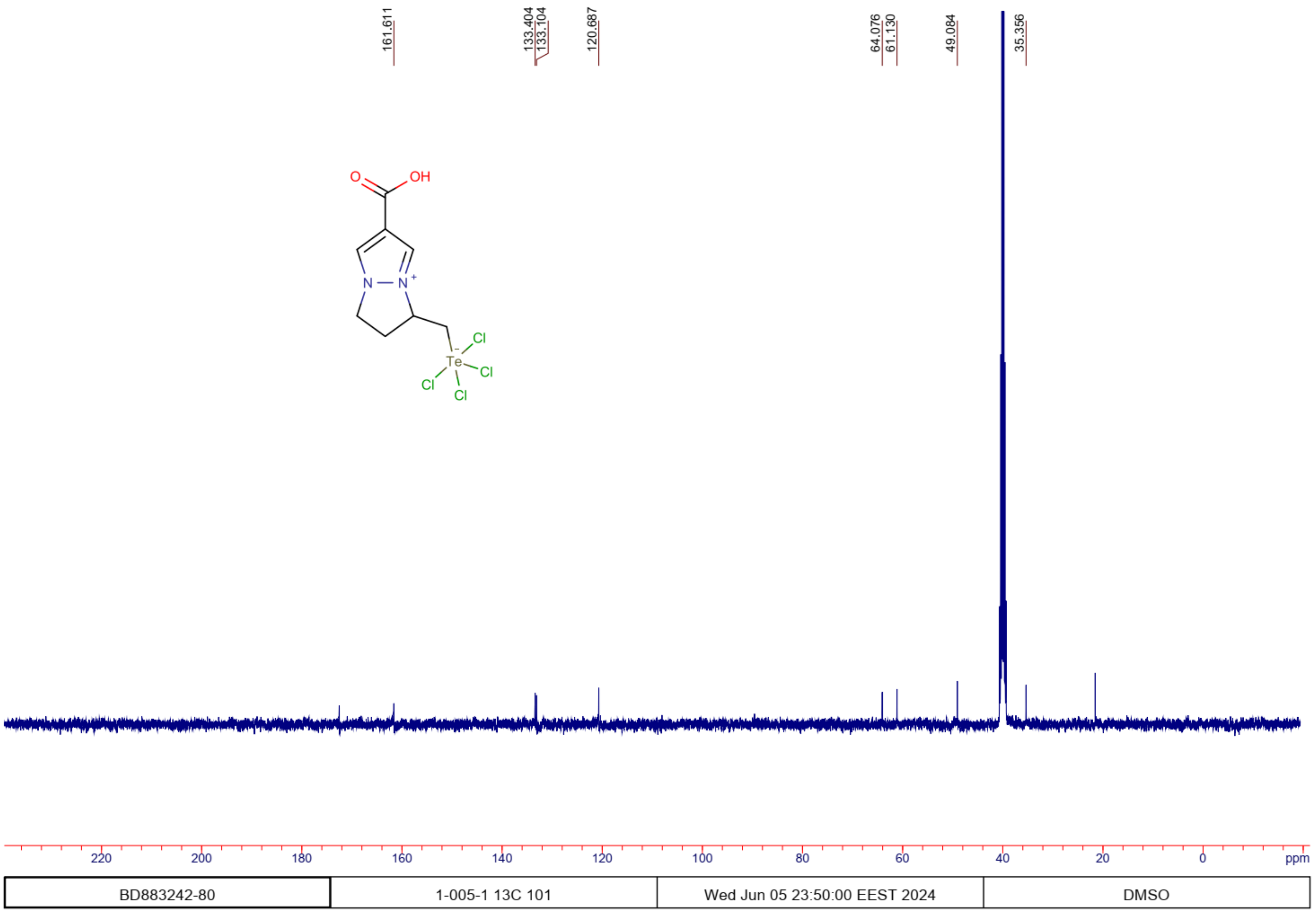

Figure S29. NMR ^{13}C compound 17
